# Extracellular Galectin 4 Drives Immune Evasion and Promotes T-cell Apoptosis in Pancreatic Cancer

**DOI:** 10.1158/2326-6066.CIR-21-1088

**Published:** 2022-12-20

**Authors:** Tommy Lidström, Joshua Cumming, Rahul Gaur, Lars Frängsmyr, Ioannis S. Pateras, Matthias J. Mickert, Oskar Franklin, Mattias N.E. Forsell, Niklas Arnberg, Mitesh Dongre, Cedric Patthey, Daniel Öhlund

**Affiliations:** 1 Wallenberg Centre for Molecular Medicine, Umeå University, Umeå, Sweden.; 2 Department of Radiation Sciences, Umeå University, Umeå, Sweden.; 3 Department of Clinical Microbiology, Umeå University, Umeå, Sweden.; 4 2nd Department of Pathology, “Attikon” University Hospital, Medical School, National and Kapodistrian University of Athens, Athens, Greece.; 5 Lumito AB, Lund, Sweden.; 6 Department of Surgical and Perioperative Science, Umeå University, Umeå, Sweden.

## Abstract

Pancreatic ductal adenocarcinoma (PDAC) is characterized by rich deposits of extracellular matrix (ECM), affecting the pathophysiology of the disease. Here, we identified galectin 4 (gal 4) as a cancer cell–produced protein that was deposited into the ECM of PDAC tumors and detected high-circulating levels of gal 4 in patients with PDAC. In orthotopic transplantation experiments, we observed increased infiltration of T cells and prolonged survival in immunocompetent mice transplanted with cancer cells with reduced expression of gal 4. Increased survival was not observed in immunodeficient RAG1^−/−^ mice, demonstrating that the effect was mediated by the adaptive immune system. By performing single-cell RNA-sequencing, we found that the myeloid compartment and cancer-associated fibroblast (CAF) subtypes were altered in the transplanted tumors. Reduced gal 4 expression associated with a higher proportion of myofibroblastic CAFs and reduced numbers of inflammatory CAFs. We also found higher proportions of M1 macrophages, T cells, and antigen-presenting dendritic cells in tumors with reduced gal 4 expression. Using a coculture system, we observed that extracellular gal 4 induced apoptosis in T cells by binding N-glycosylation residues on CD3ε/δ. Hence, we show that gal 4 is involved in immune evasion and identify gal 4 as a promising drug target for overcoming immunosuppression in PDAC.

## Teaser

Galectin 4 is a cancer cell–produced protein abundant in pancreatic tumors. The data show that galectin 4 blocks antitumor immunity and identify galectin 4 as a potential novel drug target for treating pancreatic cancer.

## Introduction

Pancreatic ductal adenocarcinoma (PDAC) is a major cause of cancer-related deaths worldwide (1), with a 5-year survival of 7% to 10% (1, 2). PDAC is characterized by a dense desmoplasia consisting of extracellular matrix (ECM) proteins, pancreatic stellate cells, and immune cells, which account for up to 90% of the tumor volume (3–5). A dense desmoplasia is also a feature of chronic pancreatitis, a risk factor for PDAC, making it in some cases difficult to morphologically distinguish it from PDAC (4). Checkpoint inhibitors have proved therapeutically effective in several cancer types (6–8), but not in PDAC (9, 10), which is thought in part to be a result of this dense stroma. Identifying and targeting immunosuppressive components of the stroma, therefore, represents an opportunity to augment immunotherapy efficiency in PDAC.

Galectins, galactose-binding lectins with a carbohydrate recognition domain, are a family of proteins that have a role in immunomodulation (11–13). Galectin 4 (gal 4) is a tandem-repeat galectin consisting of two carbohydrate recognition domains connected by a flexible linker (14). It is expressed in epithelial cells throughout the gastrointestinal (GI) tract, including the antrum, ileum, colon, and rectum (15) and has been described previously to bind glycosylated amino acid residues (16, 17).

Gal 4 exhibits a dual role in modulating immunity, having the ability to exacerbate intestinal inflammation by increasing IL6 production in CD4^+^ T cells (18). Contrastingly, in inflammatory bowel disease, extracellular gal 4 may impair inflammation by binding to the CD3 receptor on T cells, preventing cycling and expansion that eventually leads to calpain-mediated T-cell apoptosis (15). Independent of the immune-modulatory extracellular role, previous work has shown that gal 4 exhibits distinct cancer cell–intrinsic functions in PDAC. High expression of gal 4 associates with reduced migration and metastasis (19, 20), and this effect has been linked to modulation of the wnt/β-catenin pathway. Similarly, in resected PDAC tumors, low expression of gal 4 associates with recurrence and poor tumor differentiation (21).

These differences in function between intracellular and secreted gal 4 have made it difficult to fully evaluate its role in PDAC. In this study, we investigated the pathophysiological role of extracellular gal 4 in PDAC, describe for the first time the pivotal role of gal 4 in PDAC tumor-mediated immune suppression, and highlight extracellular gal 4 as a promising target for future drug development.

## Materials and Methods

### Analysis of ECM mass spectrometry data

Data from a previously published quantitative mass spectrometry data set of ECM from mouse and human pancreatic tumor samples (22) were analyzed for the highest fold-change protein expression compared with normal pancreatic tissue. All human samples were sourced from Upper GI biobank and Pathology Biobank, Umeå University Hospital. Human pancreatic tissue samples included 2 normal pancreases, surgically resected from patients with duodenal adenoma; 2 precancerous resected pancreases, pathologically diagnosed for PanIN lesions; 2 resected pancreas samples from chronic pancreatitis, and 11 tumor samples from patients with PDAC who had undergone Whipple procedure (3 well differentiated, 4 moderately differentiated, and 4 poorly differentiated tumors). Mouse pancreatic samples include 3 wild-type from C57BL6J mice, 3 each from early PanIN and late PanIN (KrasLSL.G12D/+; PdxCretg/+; KC mice), and 3 tumors (KrasLSL.G12D/+; p53R172H/+; PdxCretg/+; KPC mice). The published mass spectrometry data were retrieved from MassiIVE (http://massive.ucsd.edu), using the identifier: MSV000082639. The data were analyzed using Microsoft Excel and illustrated using GraphPad Prism.

### Human biological material and ethical considerations

Serum samples and pancreas tissues from normal, PanIN, pancreatitis, and PDAC tumors, were sourced from Upper GI (ÖGI) Biobank, Umeå University Hospital (Sweden). For control, healthy tissue samples, including liver, lymph nodes, colon, breast, and skin (one sample each), and PDAC metastasis in liver and lymph nodes (one sample each) were obtained from the Pathology Biobank, Umeå University Hospital (Sweden). Selection criteria for samples are mentioned in the relevant sections. Serum samples were collected during 2014–2019 from 23 patients with PDAC and 11 age-matched controls (no known cancer). 3 controls were excluded; 2 diagnosed with suspected gastritis, 1 diagnosed with inflammatory bowel disorder (IBD). All serum samples were freshly frozen without EDTA. The study was conducted according to declaration of the Helsinki and was approved by the ethical review board at Umeå University (09–175M and 2019–00399). All subjects taking part in the study provided written informed consent.

### IHC

Mouse tissues were fixed for at least 24 hours in 4% buffered formalin, then washed once in 70% ethanol and kept in solution until embedding. The embedded tissue was sectioned in 4-μm sections using a microtome (Leica RM2165) and was fixed onto Superfrost Ultra Plus glass slides (Thermo Fisher Scientific Inc., #J1800AMNZ) by baking at 60°C for 1 hour. Sections were deparaffinized in xylene and gradually rehydrated in 100%, 95%, and 70% ethanol. Antigen retrieval was performed in a pressure cooker (Aptum, #2100 retriever) in sodium citrate buffer (pH 6.0). For intracellular staining, tissues were permeabilized with 0.2% Triton-X 100 (Merck #11332481001) in TBS for 10 minutes before blocking. Sections were blocked in either 20% normal goat serum (Thermo Fisher Scientific, 11477119) or 2.5% normal horse serum (Vector laboratories; S-2012–50), depending on the secondary antibody, for 1.5 hours and incubated overnight with primary antibody in predetermined dilutions (Supplementary Table S1). Tissues were blocked for endogenous peroxidase with 3% H_2_0_2_ in TBS, followed by incubation in appropriate biotinylated secondary antibody [anti-mouse IgG: Vector Laboratories (#BA-9200–1.5; 2 μg/mL); anti-Rabbit IgG: Vector Laboratories (#BA-1000–1.5; 2 μg/mL)] diluted in 1% BSA (Sigma-Aldrich #A4503–50G) in TBS for 1 hour. After 2 stringent washes in TBS supplemented with 0.025% Triton-X 100, the tissue samples were incubated for 45 minutes with Elite ABC-HRP (avidin-peroxidase; Vector Laboratories #PK-7200)followed by staining with 3,3′-diaminobenzidine (DAB) substrate (Vector Laboratories #SK-4100). Mayer's Hematoxylin (HTX; Histolab Products AB #01820) was used as a nuclear counterstain.

Dako staining kit (Envision+ #K406511–2) was used to detect gal 4 in mouse short-term cohort, as well as, in human patient–derived tumor, normal, and PanIN sections, following the manufacturer's instructions. Sectioning, deparaffinization, and antigen retrieval performed as described above. Dako dual enzyme block was used to block endogenous peroxidases, followed by blocking in 2.5% normal horse serum (Vector laboratories #S-2012–50), and primary antibody (diluted in 2% BSA in TBS) incubation for 40 minutes at room temperature. Next, the sections were incubated for 30 minutes with horseradish peroxidase (HRP)–conjugated polymers (provided with the kit). The DAB substrate (included with the kit) incubation time was optimized for each primary antibody. HTX (Histolab Products AB #01820) was used as a nuclear counterstain. For gal 4, human normal sections containing liver, skin, breast, and colon were obtained from the Umeå University hospital biobank, colon sections were used as positive controls for gal 4, and for CD4, CD8, and FOXP3, lymph node sections served as positive controls. Images were scanned on a 3DHISTECH Pannoramic 250 flash III. Analysis was performed in Qupath software (23), adjusting threshold depending on section background.

### Analysis of IHC tissue sections

Tumor sections were assessed, and regions of empty space, lymphoid infiltrates, and normal tissue were removed from analysis. Tumors were annotated, annotations served as tumor area determination in the murine short-term cohort. The short-term cohort included pancreas obtained 1 to 4 weeks after orthotopic transplants of gal 4 knockdown and scramble control organoids, as described below. Images were then estimated for stain vectors that more accurately estimate the colors (here, DAB and hematoxylin) in the annotated area. Immune cell infiltration density was determined with the “fast cell count (brightfield),” with manual adjustment of thresholds for background in the section (gaussian 1.9–2.2 μm, background radius 10–15 μm, cell detection threshold 0.4–0.6, DAB threshold 0.4, detection diameter 25 pixels). All quantification was performed with Qupath v.0.1.2. For the evaluation of cleaved caspase 3 immunostaining, the number of positive cells in the epithelial compartment was counted per high-power field (magnifications, ×400). Surface epithelium in the small intestine served as the internal positive control.

### Photon-upconversion nanoparticles

Photon-upconversion nanoparticles (UCNP) are sub 100-nm particles that have several unique photophysical properties, rendering them excellent labels for IHC applications. UCNPs absorb near-infrared light and emit light of a shorter wavelength. This anti-Stokes process reduces the optical background by completely suppressing tissue autofluorescence (24). In addition to that, UCNPs are extremely photostable and do not show any signs of photobleaching, even after continuous excitation for over an hour (25). The UCNP type used in this study consisted of a hexagonal NaYF4 host lattice that was doped with Yb^3+^ and Tm^3+^. The 980-nm excitation light is absorbed by the sensitizer Yb3+ and transferred to the activator Tm3+ that emits at 810 nm. With an average size of 56 nm, the UCNPs used here were bright enough to allow for the detection of each nanoparticle in an upconversion whole-slide scanner developed by Lumito.

The tissue sections were baked for 1 hour at 60°C, followed by dewaxing in xylene (2×5 minutes) and rehydration in an increasing water/ethanol gradient (100% ethanol 2×5 minutes, 96% ethanol 5 minutes, 70% ethanol 5 minutes, distilled water 5 minutes). For heat-induced epitope retrieval, the slides were transferred into preheated EnVision Flex high pH buffer (Agilent #K8004; 95°C) and incubated for 20 minutes in a water bath (temperature of EnVision Flex high pH buffer 95°C). After 20 minutes, the container with the slides in EnVision Flex high pH buffer was removed from the water bath and allowed to cool down for 20 minutes. The slides were transferred to a Coplin jar filled with 50 mmol/L Tris/HCl, 130 mmol/L NaCl, pH 7.4 (TBS) for 1 minute and then counterstained with Mayers hematoxylin (Histolab Products AB; 01820) for 3 minutes. After washing the slides with TBS, the tissue sections were encircled with a ImmEdge Hydrophobic Barrier PAP Pen (Vector Laboratories; H-4000) and transferred to TBS. Nonspecific binding sites were blocked using TBS containing 10% Superblock TBS (Thermo Fisher Scientific, 37537), and 5% goat serum (Abcam, ab7481) for 45 minutes. The slides were washed with TBS, followed by blocking of endogenous biotin using a ready-to-use biotin blocking kit (Vector Laboratories, SP-2002). Each section was first incubated for 20 minutes with the streptavidin solution, washed with TBS (2×1 minute), and then incubated for 20 minutes with the biotin solution. The slides were washed 2×1 minute with TBS, and the primary gal 4 antibody (1:50 dilution in TBS, 5% Superblock, 5% goat serum, 0.01% Tween 20, Novus Biologicals, NBP2–48605) was applied and incubated for 1 hour at room temperature. The slides were washed 2×1 minute and a biotinylated secondary goat anti-rabbit antibody was applied (2 μg/mL in TBS, 10% Superblock, 0.01% Tween 20, Jackson Immunoresearch, 111–065–144). After incubating for 1 hour with the secondary antibody, the slides were washed 2×1 minute and incubated for 45 minutes with a streptavidin–UCNP conjugate (1:100 in UCNP dilution buffer, Lumito). The slides were washed 3×1 minute with Lumito washing buffer, mounted using Fluoroshield (Merck/Sigma-Aldrich, F6182–20ML), and sealed with clear nail polish. Slide imaging was performed in Lumito's upconversion whole-slide scanner.

### Analysis of upconversion nanoparticle staining

For the analysis of UCNP labeling, 5 tumor glands were selected at least 200-μm apart from adjacent tissue to avoid signal spillover from a human moderately differentiated tumor. Control sections containing liver, skin, breast, and colon tissue were obtained from the Pathology Biobank, Umeå University Hospital (Sweden), and used as controls for the staining. Tumor sections were annotated with 100-μm outer borders. Sections were separated into 25 μm^2^ tiles, and between 5 and 10 tiles were selected for epithelial, proximal, and distal areas to the tumor. Signal was quantified using the compute intensity feature in QuPath, and the average signal for each category was calculated for all 5 tumors.

### The cancer genome atlas data sets

The human pancreatic cancer (PAAD) The Cancer Genome Atlas (TCGA) RSEM normalized RNA sequencing (RNA-seq) data set (26) was downloaded from the xenabrowser website (27). Of the 176 patient samples in the PAAD data set, 74 patient samples with PDAC diagnosis and moderate differentiation grade (inclusion criteria) were included in the main paper analysis, and all 176 patients were included in a separate analysis presented in the Supplementary Material (all patients with verified PDAC diagnosis). The TCGA RSEM normalized RNA-seq colon cancer (COAD) data set was also downloaded from the xenabrowser website (27). Because no differentiation data were available in the COAD data set, all patients were included in the analysis. In addition, breast cancer, stomach cancer, bladder, cervical cancer, lung cancer, and prostate cancer RSEM normalized RNA-seq data sets were downloaded from the xenabrowser website (27). The breast cancer, stomach cancer, bladder, cervical cancer, lung cancer, and prostate cancer data sets were analyzed for *LGALS4* expression in tumors by graphical illustration using GraphPad Prism.

### *LGALS4* survival analysis

For the PAAD TCGA data set (26), Kaplan–Meier survival analysis was performed on all patients, and subset of patient with moderate differentiated tumors, in R using the survival package (v3.2–7; Therneau, 2020; ref. 28). To identify high- and low-expressing groups, Z-scores were calculated for each patient based on RSEM normalized transcript counts for *LGALS4*. Z-scores are a numerical quantification of the deviation from the mean in terms of standard deviations. Z-scores for *LGALS4* were calculated as follows: Z = (*x* – μ) / σ, where *x* = normalized gene count, μ = mean gene count, and σ = standard deviation. To determine the cutoff value for high and low groups, a Cox proportional hazards regression model was fitted, and log-rank scoring used to assess *P* values at different cutoff value percentiles (0.2–0.8) for patient Z-scores using the survival package. The percentile where the largest difference in survival between the high (>percentile) and low groups (<percentile) was selected.

### Cytolytic effect and CD8^+^ T-cell activation scoring using the COAD and PAAD TCGA data sets

Gene signatures for cytolytic effect and CD8^+^ T-cell activation score in Supplementary Table S2 were obtained from Azizi and colleagues (29). Z-scores for individual genes in the signature were calculated as described above, and the mean of all composite gene Z-scores for a gene signature gave the signature score. Gene signature scores were compared between patients in the *LGALS4* high and low-expression groups as described in moderately differentiated PDAC cohort and the full COAD data set.

### ELISA on blood sera

Patient serum samples were diluted in ELISA sample plasma diluent (Bio-Rad, OBT1998GX; 1:10 1:50 and 1:100, if multiple dilutions were present in the standard curve range the lowest dilution was used. Controls were sample diluent only. Gal 4 concentration was subsequently determined using an ELISA kit (R&D Systems, DY1227–05) according to the manufacturer's instructions. Standard curve was part of the kit. Concentrations were calculated using GraphPad Prism with interpolation of a linear standard curve. Spectramax i3x (Molecular Devices) was used for optical density (OD) measurements at 450 and 540 nm, in triplicate. Physical imperfections in the plate were corrected by subtracting OD at 540 nm from that at 450 nm. Average of triplicate samples was used for quantification.

### Mice

C57BL6J and Rag1^−/−^ mice were obtained from The Jackson Laboratory, and housed in Umeå University UCCB facility. Ethical approval was acquired from Swedish Board of Agriculture (A39–2017), and animals were housed according to Swedish rules on research and conducted in accordance with the Institutional Animal Care and Use Committee (IACUC) at CSHL. KrasLSL.G12D/+; p53R172H/+; PdxCretg/+ (KPC) mice were bred in-house by pairing heterozygous KrasLSL.G12D/+; p53R172H/+ (KP) mouse with homozygous transgenic Pdx1-Cre (C) mouse. KP mouse were, in turn, bred by pairing KrasLSL.G12D/+ mouse with p53R172H/+ mouse. KP and C mice strains were obtained from The Jackson laboratory.

### Cell lines

R26 cells were obtained from a KPC mouse tumor. 293T cells were obtained from the ATCC (CRL-3216). Organoid-derived 2D cell lines [R26 clone 1, gal 4–knockout (KO) exon 2 clone1, 4 and 5] and organoid cell lines [gal 4–knockdown (KD) and scramble controls, T3, T5, T8 and T9] were kindly provided by Professor David Tuveson at Cold Spring Harbor Laboratory. Gal 4–KO and control R26 cells were generated using CRISPR/Cas9 as indicated below. Before use, all KPC-derived cell lines were genotyped to confirm the heterozygous status in Kras(G12D) and p53(R172H) genes. All cell lines were intermittently tested (in-house) for *Mycoplasma* contamination (most recent test for all 2D and organoid cell lines was 220513) for the duration of the project using 16s rRNA-specific primers. IDEXX Impact IV sterility tests were also performed for cell lines used in the orthotopic transplantation experiments.

### Establishing monolayer and organoid cell lines from KPC mouse model

Tumors were excised from the KPC mouse model when they reached 8 to 10 mm (3–6 months of age) and kept on ice. Tumors were minced and digested for 8 to 18 hours in a rotating shaker with collagenase XI (0.12 mg/mL; Sigma #7657), dispase II (Thermo Fisher Scientific, #17105041) in DMEM (Thermo Fisher Scientific #12634028) supplemented with 1% FBS (Gibco #10270–106) and 1% pen-strep (100X stock; Thermo Fisher Scientific #15140122). The tissue was further digested in TrypLE Express (Gibco, #12605010) and DNaseI (0.01 mg/mL, Sigma, #D5025–150KU) for 10 minutes. Digests were centrifuged and resuspended in 100% Matrigel (Corning #356231). Cells were cultured in complete mouse organoid feed media (30) containing Advanced DMEM/F-12 (Thermo Fisher Scientific #12634028), 1×1M HEPES (diluted to 10 mmol/L; Gibco, #15630056), 1X penicillin/streptomycin (100X stock; Thermo Fisher Scientific #15140122), 1X GlutaMAX Supplement (Gibco, #35050061), 0.5 μmol/L (0.21 μg/mL) A83–01 (TGFβ inhibitor; Tocris, #2939), recombinant murine EGF (mEGF; 0.05 μg/mL; Gibco, #PMG8041), recombinant FGF (FGF-10; 0.1 μg/mL; Peprotech, #100–26–250 μg), 0.01 μmol/L (0.021 μg/mL) Gastrin I (Tocris, #3006), 0.1 μg/mL, recombinant mNoggin, 1.25 mmol/L (0.2 mg/mL; peprotech #250–38–250ug), N-acetylcysteine 10 mmol/L (1.22 mg/mL; Sigma, #A9165–5g), nicotinamide 10 mmol/L (Sigma, #N0636–100G), 1X B27 supplement (Thermo Fisher Scientific, #17504044), 10% R-spondin-conditioned media (produced in-house from a cell line expressing recombinant R-spondin: Trevigen Cat # 3710–001–01) based on the method described in ref. (30), Y-27632 10.5 μmol/L (3.38 μg/mL; Rho kinase inhibitor; Sigma, #Y0503–5 mg). Established organoids were formed after 3 to 5 days. Organoids were dissociated into single-cell suspensions as described below, and then plated (800,000 cells in 3 mL media) on tissue cultured treated plastic (Sarstedt #83.3920) with high-glucose DMEM (#Sigma D5796) containing 5% FBS and 1% penicillin/streptomycin to generate 2D cell lines.

### Culturing conditions of murine cell lines

KPC tumor cells (2D) were cultured in high-glucose DMEM media (Sigma #D5796) containing 5% FBS and 1% penicillin/streptomycin. KPC-derived organoids were cultured in murine feed media (31). To passage cells, Matrigel domes were dissolved using ice-cold triple plus media: Advanced DMEM/F-12 (Thermo Fisher Scientific #12634028), 1×1 mol/L HEPES (diluted to 10 mmol/L; Gibco, #15630056), 1X penicillin/streptomycin (100X stock; Thermo Fisher Scientific #15140122), 1X GlutaMAX Supplement (100x stock, Gibco, #35050061). Organoids were then mechanically dissociated using fire polished pipettes and seeded into fresh Matrigel. Passaging was performed at a ratio of 1:4 or was dependent on density of cells. Cells were undergone 12 passages before use. When thawed from frozen stock, complete mouse organoid feed media (defined above) supplemented with 10.5 μmol/L (3.38 μg/mL) Y-27632 (Rho kinase inhibitor) for the first passage. All cells were cultured at 37°C with 5% CO_2_.

### Generation of CRISPR/cas9 gal 4–KO cell lines

To generate R26 control and gal 4–KO cell lines, MSCV-Cas9-puro (Addgene plasmid # 65655), a gift from Christopher Vakoc (Cold Spring Harbor Laboratory), were transfected into 293T cells together with psPAX2 (Addgene #12260) and pMD2.G (addgene #12259) to produce lentivirus. KPC-derived 2D tumor cell lines were infected with the lentivirus and selected by puromycin (1 μg/mL; Sigma-Aldrich #P8833) for 2 days to generate cell lines with stable CAS9 expression. mRNA sequences for gal 4 were acquired from NCBI (NM_006149.4), and guides were constructed against the first and second exon using the CRISPR design tool at http://crispr.mit.edu. Guide RNA against gal 4 targeting exon 1 (CACCGGTGGGCTGGTAGCCCGGTGC, AAACGCACCGGGCTACCAGCCCACC) and exon 2 (CACCGAAACGGACATCCCGACACTG, AAACCAGTGTCGGGATGTCCGTTTC) were constructed and cloned into LRG vector with GFP (Lenti-sgRNA-EFS-GFP; Addgene #65656). Next, the target vector was transformed into Stbl2 competent cells and grown on agar plates. To verify successful transformation, 6 clones were sequenced with the pU6 forward sequencing primer (ACTATCATATGCTTACCGTAAC). Small guide (sg)RNA-GFP lentivirus was produced in 293T cells, with media changed after 24 hours and collected after an additional 24 hours. Lentivirus-containing media were filtered and used to infect CAS9-expressing KPC-derived 2D cells. In addition, sgRNAs directed against Replication Protein A3 (RPA3; GACCGGCCCGTGTGCTTCGTGGG), were cloned and amplified as described above to generate RPA3-LRG-GFP lentiviruses that were used to infect MSCV-Cas9 cell lines. The RPA3-transduced cell line was then used as a positive control in the proliferation competition assay. To verify the successful infection, cells were sorted on the basis of GFP positivity in FACSAria cell sorter (BD Biosciences), and a genomic cleavage detection kit (Geneart #A24372) was used with designed primers against exon 1: (fw: GAAGTCTCCACCAGAGAGACC, rv: CTGTCTCACCGTCTCATGTTCTC) and exon 2 (fw: CTCATCTCCCCCGCTTAAGG, rv: CCCAGTCATAGAGCCACCTC). Cell lines with sgRNAs directed against gal 4 exon 1, exon 2, and RPA3 were used in bulk in competition assays as described below.

Cells were grown for 23 days in a 6-well plate in high-glucose DMEM media (Sigma #D5796) containing 5% FBS and 1% penicillin/streptomycin, and GFP fluorescence intensity was measured with flow cytometry (Millipore Guava EasyCyte HT) over 23 days and normalized against itself and R26 control cells. GFP-positive cells were in-parallel single-cell sorted into individual wells of a 96-well plate (Corning #3603) and expanded into separate cell lines over 2 weeks in high-glucose DMEM media (Sigma #D5796) containing 5% FBS and 1% penicillin/streptomycin. The remaining cells were bulk-sorted on GFP positivity, expanded as described previously, and used to determine proliferation rate. Cells were seeded at a density of 3,000 to 5,000 cells per well in a 96-well plate (Corning #3603) in high-glucose DMEM media (Sigma #D5796) containing 5% FBS and 1% penicillin/streptomycin containing 5% FBS. GFP fluorescence was measured every day for 5 days using a Spectramax i3x (Molecular Devices) and normalized to fluorescence recorded at the 5-day endpoint (100%).

### Sequencing validation of CRISPR/cas9-modified gal 4–KO cell lines

Genomic DNA was extracted (Qiagen #69504) from gal 4–KO and R26 control cell lines. The exon 1 region of the *Lgals4* gene was amplified by PCR (Applied Biosystems, VERITI) with the following primers: Fwd 5′-GAAGTCTCCACCAGAGAGACC-3′ and rev 5′-CTGTCTCACCGTCTCATGTTCTC-3′. The PCR product was sequenced using the Sanger method (provided by Eurofins Genomics) in forward and reverse directions using the same primers. The electropherograms were analyzed manually to determine the sequence of both alleles, and the inferred sequences were aligned to the corresponding mouse reference genome (accession number GCA_000001635.9) using MAFFT (https://mafft.cbrc.jp/alignment/server/).

### Generation of gal 4–KD 3D organoid cell lines

To generate scramble control and gal 4–KD 3D organoid cell lines, glycerol stocks of Stbl2 cells expressing 5 different short-hairpin (sh)RNA against the gal 4 sequence in pLKO.1 vector with puromycin resistance (Merck, #SHCLNG TRCN0000066378 - TRCN0000066382) or a scramble sequence, targeting no known mammalian genes, in the same vector (Merck #SHC002) were amplified, and lentivirus–hairpin–puroR were generated as described above. Confluent organoids were dissociated to single-cell suspensions as specified above, and tumor cells were infected with the lentivirus-target and selected by puromycin (1 μg/mL) for 2 days to generate cell lines with stable expression of the scramble sequence or short-hairpin directed against gal 4. Organoids were transduced with 5 different hairpins: TRCN0000066378 (CGGAAATTCCTTCTATGAATA), TRCN0000066379 (CCGAAGAACCATCATAATCAA), TRCN0000066380 (CCTGGAACTTCAGTCCATCAA), TRCN0000066381 (ACACGATGCAAAGTGGACAAT), and TRCN0000066382 (GAGCCATGACTATTCCAGCTT), referred to as sh78-sh82 in text, or scramble shRNA (Addgene plasmid #1864; clones can be found at: https://portals.broadinstitute.org/gpp/public/). To confirm similar proliferation of the generated cell lines, a CellTiter-Glo 2D cell viability assay (PROMEGA #G7571) was performed according to the manufacturer's instructions. Luminescence of proliferating cells was measured at 0, 72, and 120 hours (Spectramax i3x, Molecular Devices).

### Orthotopic transplantation

To assess the effect of gal 4 expression *in vivo*, we performed transplantation experiments with 2D cell lines, R26 control, and gal 4–KO, orthotopically into female or male C57BL6J mice ages between 42 and 52 days. The mice were randomly selected for inclusion, independent of the age. Mice were sedated using isoflourane (Attane vet 1,000 mg/g ELÄMILLE VNR 17 0579) at 4% concentration, and 2.5×10^5^ R26 control or gal 4–KO cells were suspended in triple plus media. 50 μL were transplanted into splenic lobe (tail) of the pancreas. Incisions into the peritoneum was sutured (Vicryl Rapide 6–0 AgnTho's V32H), skin was closed with Michel Suture Clips (7.5–1.75-mm AgnTho's 12040–01).

Orthotopic transplants were included in the study if no leakage occurred during transplantation, a clear injection bubble formed, and no excessive bleeding took place during injection or surgery. Following surgery, the general health status of transplanted mice was monitored according to ethical guidelines. Mice were injected with one dose per day of carprofen (Norocarp vet, VNR 027636), 20 mg/kg/d, as analgesic for a total of 3 days following surgery. The mice were assessed for pain, body weight, and temperature to determine general health status. At 26 to 28 days mice were sacrificed, necropsy performed, and tumors and spleens collected.

For survival and short-term experiments, organoid transplantations (gal 4–KD or scramble controls) were performed in C57BL6J or RAG1^−/−^ mice using 2.5×10^5^ cells (or 5×10^5^ cells if used for single-cell sequencing experiments described below) suspended in 30% Matrigel (Corning #356231) in triple plus media (defined above). The transplantation procedure was performed as described above. Surgeries were performed in an alternating fashion, transplanting 1 KO/KD cell line, followed by transplantation of 1 control cell line. Mice for the short-term experiments (short-term cohort) were sacrificed after 1, 2, and 4 weeks. For the survival experiment (survival cohort), mice were monitored daily and were sacrificed when moribund. At necropsy, the tumors and remaining normal pancreas was collected.

### ISH

Freshly prepared formalin-fixed paraffin-embedded KPC tumor tissues were sectioned (6-μm thickness) and analyzed for gal 4 (*Lgals4*), cytokeratin 18 (*Krt18*), and fibroblast-activation protein (*Fap*) expression using the ViewRNA ISH Tissue 2-Plex Assay (Affymetrix #QVT0012), with probes against gal 4 (VB1–18194–01), cytokeratin 18 (VB1–16010–06), and FAP (VB6–17016–06), according to the manufacturer's instructions. In short, tissue was deparaffinized in xylene and washed and rehydrated in ethanol. The sections were pretreated with heat for 10 minutes at 90°C to 95°C in the provided buffer, followed by digestion with protease solution included in kit for 10 minutes at 40°C and then fixed in 10% normal-buffered formalin included in kit, followed by hybridization with the target probes against *Lgals4, Fap*, and *Krt18* for 2 hours at 40°C. The sections were stored overnight in storage buffer (included in kit) before proceeding with signal amplification and detection. The sections were pre-amplified in reagents included in the kit for 25 minutes at 40°C in a HybEZ hybridization oven (Advanced Cell Diagnostics), followed by amplifier hybridization using reagents included in kit for 15 minutes at 40°C, followed by incubation with label probes (included in kit) at 40°C for 15 minutes, and the substrate (included in kit) was then added. The blue label probe was applied first, with subsequent fast blue substrate incubation, followed by red probe hybridization and application of the red substrate. Sections were then counterstained in Gil's hematoxylin and dipped in 0.01% ammonium hydroxide. Slides were mounted with Prolong gold antifade mountant (Thermo Fisher Scientific). The images were captured on a Nikon eclipse E600 microscope with a DXM 1200 camera with a ×100 objective in oil immersion using NIS-Elements F version.2.3.

### Flow cytometry analysis of orthotopic transplants

For flow cytometric analysis of tumors generated by orthotopic transplantation, the tissues were finely minced and digested in high-glucose DMEM media (Sigma #D5796) containing 10% FBS (Gibco 10270–106), 1% penicillin streptomycin (Gibco 15140–122), DNase I (0.2 mg/mL; Sigma # D5025), liberase DL (0.5 mg/mL; Sigma # 5466202001), and collagenase (2.5 mg/mL; Sigma #11088866001) for 45 minutes at 37°C with mild shaking. Following digestion, the solution was strained through a 100-μm cell strainer and washed with FACS buffer (2% FBS in PBS). Following centrifugation, cell pellets were dissolved in ACK lysis buffer (Thermo Fisher Scientific, A1049201) for 5 minutes on ice to lyse red blood cells. Cells were counted, and viability was determined before proceeding with immune cell and apoptosis panel staining. Up to 1×10^6^ cells were blocked with anti-CD16/32 (BioLegend# 101302) for 10 minutes, followed by antibody staining with immune cell panels in predetermined concentrations (Supplementary Table S2) for 30 minutes on ice. To determine viability, cells were stained with live/dead fixable red stain (Thermo Fisher Scientific # L34971) for 30 minutes on ice. For intracellular staining, cells were fixed in 4% paraformaldehyde, followed by permeabilization with 0.1% saponin (Sigma-Aldrich #47036–50G-F). To determine apoptosis, the Annexin V–7AAD apoptosis detection kit (BioLegend # 640930) or 7-AAD Viability Staining Solution (Nordic Biosite #420404) was used following the manufacturer's instructions. Stained samples were acquired on BD LSRII or Bio-Rad ZE5 and analyzed with FLOWJO (v10) software. Compensation profiles of all antibodies used were prepared using a combination of cells and UltraComp eBeads (Thermo Fisher Scientific, # 01–2222–42). Gating of multi-color panels for markers with low expression was determined using FMO (fluorescence minus one) stainings. Data were included with successful enzymatic dissociation and successful staining of intratumoral immune cells. R26 tumors were used as controls for CD8^+^ T-cell polarization.

### Western blotting

Organoids were harvested in Cell Recovery Solution (Corning Cell Recovery Solution, 100 mL #354253) and incubated with rotation for 1 hour at 4°C. Cells were then pelleted, and lysed in 0.1% Triton X-100, 15 mmol/L NaCl, 0.5 mmol/L EDTA, 5 mmol/L Tris, pH 7.5 supplemented with protease Mini-complete protease inhibitors (Roche) and a phosphatase inhibitor cocktail (PhosSTOP; Roche). Cells were incubated on ice for 30 minutes. Protein lysates (10 μg of protein) were separated by SDS-PAGE, transferred to a polyvinylidene difluoride membrane (Bio-Rad #1704274), blocked with 5% BSA in TBST (1% Tween 20; Sigma-Aldrich #P9416; TBS), and incubated with primary antibodies overnight at 4°C. Proteins were detected using HRP-conjugated secondary antibodies (Thermo Fisher Scientific, #A15999: ABCAM #ab6721).

### Splenocyte isolation

Spleens were collected from C57BL6/J mice, finely minced, and cells were suspended in PBS containing 2% FBS. Following centrifugation, the pellet was resuspended in ACK lysis buffer to lyse red blood cells for 5 minutes on ice. Cells were washed and counted before use in downstream applications.

### Flow cytometry sorting for RNA-seq of the gal 4–KD orthotopic model

Tumors from orthotopic transplantations of gal 4–KD or scramble control organoid lines (T5 line) were collected, dissociated as indicated above, and blocked with anti-CD16/32 as described previously. Cells were stained with anti–Epcam-PE (Thermo Fisher Scientific, #12–5791–82, 1:150), anti–Pdpn-biotin (Thermo Fisher Scientific, # 13–5381–82, 1:100), and anti–CD45-FITC (Thermo Fisher Scientific, #11–0451–82, 1:100) on ice for 30 minutes. Cells were also stained in 1 μg/mL DAPI (Roche # 10236276001) for live/dead discrimination. DAPI^−^ (live) cells were sorted with a BD Aria-III sorter.

### Single-cell RNA-seq

Pdpn^+^ CAFs and CD45^+^ immune cells were collected in chilled 2% FBS in PBS. The cells were washed and resuspended in 0.2% BSA in PBS at a concentration of 1,000 cells/μL before pooling equal numbers of Pdpn^+^ and CD45^+^ cells. A total of 7,500 cells were loaded per well into a 10x Chromium cartridge (10x Genomics # 1000127), and cDNA and libraries were prepared according to the manufacturer's instructions (Chromium Single Cell 3′ dual indexing v3.1 protocol). The libraries were sequenced in-house with Illumina Nextseq 500/550 v2 reagents to a depth of 50 to 60 thousand reads per cell, with 28 bp for read 1 and 44 bp for read 2. The raw reads were processed with the Cell Ranger pipeline version 6.1.2 with default parameters (10x Genomics, freeware), mapping the reads on the refdata-gex-mm10–2020-A reference genome, yielding 7,677 single cells. The data were processed with Seurat version 4.1.0 (32). Briefly, cells were filtered for unique molecular identifiers (UMI) count per cell (2,000<nCount_RNA<50,000), genes detected per cell (800<nFeature_RNA<8,000), and the percentage of mitochondrial reads (percent.mt<10). The data were normalized with the LogNormalize function and highly variable genes were selected using Seurat's Find Variable Features function with the “vst” method and default parameters. Cells from the scramble control and gal 4–KD libraries were integrated using the Integrate Data function, including 50 dimensions and otherwise keeping default settings. Clustering was performed with default methods (Find Neighbors function with dimensions 1:30, Find Clusters function with resolution = 0.2). Clusters identified as CAFs or immune cells were separated, and the integration and clustering were reiterated on CAFs or immune cells individually. Clusters representing cross contamination of CAFs and immune cells were identified and excluded, yielding 3,811 CAFs and 1,618 immune cells. The raw UMI counts are available on the gene expression omnibus (GEO) with accession number GSE215389. Metadata and cell annotation for the 7,677 cells are available in Supplementary File S1. Clusters of CAFs and immune cells were annotated using lists of markers derived using the Find Markers function with default parameters comparing each cluster versus all others. The top 30 genes for each cluster are listed in Supplementary Files S2 and S3. The proportion of a subtype was calculated as 100*n/*N*, where *n* is the number of cells in the cluster for the given shRNA group (scramble or gal 4–KD) and *N* is the total number of CAFs or immune cells for the given shRNA group. Signature scores were calculated as the mean of Z-scores through the genes included in the signature. The Z-score for a gene was defined as *Z* = (*X*−*M*)/SD, where *X* is the log-normalized UMI count, *M* is the mean, and SD is the standard deviation of the log-normalized UMI count for the gene, respectively. Violin plots showing normalized expression were generated in Seurat. The gene lists used as signatures are given in Supplementary File S4. All plots were made in R version 4.1.1 using the Seurat version 4.1.0 and ggplot2 version 3.3.5 packages.

### Bulk RNA-seq

Organoids from gal 4–KD or scramble control lines (T5 line) were dissociated with TrypLE, DNase I, and dispase as described above. Cells were stained and sorted using the same protocol as for tumors from orthotopic transplantation. 500 Epcam^+^ cells per sample were sorted directly into 5.5 μL of lysis buffer containing 0.2% triton and 2 U/μL RNase inhibitor (Takara Bio #2314B) before freezing on dry ice. The samples were thawed, and RNA-seq libraries were generated using the Smart-seq2 protocol according to the original publication without modifications (33). The libraries were sequenced with Illumina Nextseq 500/550 v2 reagents to a depth of 6–27 million reads per sample. Read length was single-end 75 bp per read. Adapters were trimmed using Cutadapt (34). Ribosomal (r)RNA reads were removed by mapping the reads to 5S (accession number_NR_030686.1) and 45S (accession number NR_046233.2) rRNA sequences using bowtie2 (35). The trimmed reads were aligned to the mouse reference genome using STAR (36). The GRCm39 genome assembly was used (accession number GCA_000001635.9) with the Ensembl release 104 annotations. Reads mapping to protein-coding genes were counted using HTSeq (37). Mapped reads were normalized by two different algorithms: TPM (transcripts per million) and RLE (relative log expression) as part of the DEseq2 package (38). Statistical significance of *Lgals4* downregulation in the gal 4–KD samples was tested with default settings in DESeq2. The raw and normalized read count data (TPM) can be found in Supplementary File S5.

### *Ex vivo* experiments

R26 control or gal 4–KO tumor cells were seeded on a 24-well plate (20 *k*/well) either in Matrigel domes or on a 2D surface in high-glucose DMEM media (Sigma #D5796) media with 5% FBS and allowed to adhere for 24 hours. Organoid cells were seeded in Matrigel in a 24-well plate for 24 hours in complete murine-feed media. Splenocytes were isolated from C57BL6/J mice aged between 42 and 52 days and (2×10^5^ cells/well) were added to the liquid phase in RPMI-1640 (Sigma-Aldrich #R8758) with 10% FBS, 1.5% HEPES, supplemented with 20 IU of IL2 (R&D systems 402-ML-020), 50 μmol/L 2-mercaptoethanol, and mEGF (0.1 μg/mL), T-cell media (TCM). In 3D organoid experiments, either isotype IgG2 (Bio X Cell #BE0089; 1 μg/mL) or anti–PD-1 (Bio X Cell #BE0273; 1 μg/mL) was added to the media. Every 48 hours, cells in liquid phase were collected, centrifuged, and fresh media were added. For the collection of cells, Matrigel domes were washed once with PBS, followed by dissociation of the Matrigel domes as described above.

### 2D cell assays

Splenocytes from two C57BL6/J mice were isolated as described above. 2×10^5^ splenocytes were added to R26 control or gal 4–KO cell cultures. Experiments were repeated twice with two clones of the gal 4–KO cell lines. The 2D *ex vivo* assay was performed as illustrated in Fig. 6A. Supernatants from the cocultures, containing immune cells, were collected whereas adherent cells were dissociated with TrypLE express (Thermo Fisher Scientific, 12–605–010) for 5 minutes at 37°C. Dissociated cells were added back to the collected supernatant before flow cytometric analysis. For imaging, GFP-positive 2D cells were cocultured with immune cells stained with 1 μmol/L Deep Red cell tracker solution (Thermo Fisher Scientific, CellTracker Deep Red Dye #C34565) for 30 minutes at 37°C in RPMI-1640 serum-free media. Images were captured with a Zeiss LSM 710, 63x objective/1.4 oil immersion, NA = 0.09, at room temperature using Zen 2.3 (black edition).

For CD3 chain blocking experiments, 2.5×10^5^ of both of R26 and gal 4–KO cells were seeded in TCM (timepoint 0 hours). Splenocytes were harvested as described above, and CD8^+^ T cells were purified using the EasySep Mouse CD8^+^ T-cell Isolation Kit (STEMCELL, #19853). T cells were activated for 24 hours in TCM with 2 μL of Dynabeads Mouse T-Activator CD3/CD28 (Thermo Fisher Scientific, #11456D). At timepoint 21 hours, CD3 chains fragments of CD3ε/δ (Thermo Fisher Scientific, #17126221) and CD3ε/γ (Thermo Fisher Scientific, #17146231) or BSA as a control were added at 60 μg/mL concentration. Activated CD8^+^ T cells were labeled with 2 μmol/L CFSE solution (CellTrace CFSE Cell Proliferation Kit Protocol (Thermo Fisher Scientific, #C34554), according to the manufacturer's instructions and added to the tumor cell cultures at 24 hours. After 24 hours, supernatants containing immune cells were collected. Cells were washed and stained with 7AAD and Annexin V and CFSE^+^7AAD^−^ cells were assessed for Annexin V signal. Stained samples were acquired on Bio-Rad ZE5 and analyzed with FLOWJO (v10) software.

### 3D organoid cells

Isolated splenocytes were also stained with 2 μmol/L CFSE solution, as described above. Following completion of experiment, the coculture of organoid cells and immune cells were collected from domes in cell recovery solution (Corning Cell Recovery Solution, 100 mL #354253) and left on ice for 30 minutes and were then digested in TrypLE and DNaseI (10 μg/mL) for 10 minutes at 37°C. Dispase (2 mg/mL) was added in equal volume as TrypLE and DNase solution and digested for an additional 10 minutes, to obtain a suspension of organoid-derived tumor and immune cells. These tumor and immune cells were added to a tissue culture-treated, 96-well plate for flow cytometry staining and analysis. Cells were mechanically disrupted and filtered through a 70 μmol/L strainer before flow cytometry analysis. Tumor cells and splenocytes were differentiated by CFSE positivity, and apoptosis was determined by 7-AAD positivity.

### RNA isolation

Tumor tissue from R26 or gal 4–KO orthotopic transplants, and Matrigel domes were dissolved in TRizol (Thermo Fisher Scientific, #15596018) and stored at -80°C until RNA extraction. RNA extraction was performed according to the manufacturer's instructions. In short, chlororform was added to the samples in phase-maker tubes (Thermo Fisher Scientific, #A33248). Samples were centrifuged, and the aqueous phase was transferred, followed by isopropanol addition to precipitate nucleic acids. Samples were incubated for 10 minutes at room temperature and centrifuged for 12,000 x *g* for 10 minutes. Samples were washed in 70% ethanol and resuspended in RNAse-free water. For tumor tissue samples, an additional column purification was performed to ensure good purity (Purelink RNA mini-kit, Thermo Fisher Scientific, #12183020). Concentration was determined by Qubit fluorescence measurement (Qubit 4 Fluorometer #Q33238; Thermo Fisher Scientific, #Q32855), and RNA was used for RT-qPCR.

### RT-qPCR

400-ng RNA was used to reverse transcribed cDNA using the Thermo Fisher Scientific RevertAid H Minus Reverse Transcriptase (#EP0452) according to the manufacturer's instructions. cDNA was diluted 1:4 before proceeding with RT-qPCR reactions. All primers are listed in Supplementary Table S3. Primers were stored at 100 μmol/L concentration before diluting 1:20 for use. RT-qPCR reactions were performed using PowerUp SYBR Green Master Mix (Thermo Fisher Scientific, A25777) according to the manufacturer's instructions. In short, mastermixes for the different genes were made and added to a 96-well plate. A separate plate was prepared with diluted cDNA. Mastermixes and diluted cDNA were loaded into a 384-well plate using a pipetting robot (Beckman-Coulter). The reaction was run in a Quantstudio 6 Flex system. A melting curve was recorded to verify specificity of primers. Gene expression was normalized to *Hprt* (fw: CTTCCTCCTCAGACCGCTTTT, Rv: CATCATCGCTAATCACGACGC). Gene expression was determined with 2^−ΔΔCT^ using QuantStudio Real-Time PCR Software v.1.3.

### Expression and purification of human recombinant gal 4

Human recombinant gal 4 was produced at Protein Expertise Platform at Umeå University, was used. For this, *Homo sapiens* gal 4 (*LGALS4*; NM_006149.4) mRNA, cloned into plasmid pcDNA3.1-C-(k)DYK, was purchased (Genscript # OHu15835). An N-terminal Hisx6 +TEV-site was introduced by cloning *LGALS4* into pET-His1a vector (NcoI + Acc65I). The cloning was done using Phusion polymerase (Thermo Fisher Scientific, # F631S), and the constructs were transformed into Bl21(DE3) competent cells. Cloning was confirmed by sanger sequencing (Eurofins Genomics). Expression was induced using Rosetta (DE3), auto-induction media, at 20°C for 16 hours. Bacterial cells were harvested, and lysis was performed by sonication (IKA-Werke GmbH). Protein was affinity purified using NiNTA (Thermo Fisher Scientific, # R90115), followed by tag removal using TEV-protease (Thermo Fisher Scientific, # 12575015). Both the Hisx6-TEV-tag and TEV-protease were removed by a second NiNTA run.

### Surface plasmon resonance

CM5 sensor chips and an amine-coupling kit were purchased from Cytiva (#BR100050). All surface plasmon resonance (SPR) experiments were performed at 25°C in 10 mmol/L phosphate buffer (pH 7.4), 140 mmol/L NaCl, 0.27 mmol/L KCl, and 0.05% Tween 20 as running buffer. Data were collected with a Biacore T-200 instrument at a rate of 1 Hz. Mouse CD3ε/δ (Thermo Fisher Scientific, #17146231), human CD3ε/δ (ACROBiosystems, CDD-H52wa-100 μg), and human CD3ε/γ (ACROBiosystems, CDG-H52W9–50UG) were coupled to individual CM5 sensor chips by amine-coupling reactions at pH 4 (according to the manufacturer's instructions) to an immobilization density of 3,900, 9,800, and 8,900 response units (RU), respectively. The surface of the upstream flow cell was used as a reference and was subjected to the same coupling reaction but in the absence of protein. To determine gal 4 binding, a gal 4 analyte (human recombinant gal 4) was serially diluted in running buffer in a 2-fold concentration dilution (16 to 0.5 μmol/L), and then injected over the reference and experimental surfaces for 120 seconds and a dissociation time of 120 seconds at a flow rate of 30 mL/min. Blank samples containing only running buffer were also injected under the same conditions to allow for double referencing. After each cycle, the biosensor surface was regenerated with a 60-s pulse of 10 mmol/L Tris-glycine (pH 1.5) at a flow rate of 30 mL/min. Blocking experiments were performed by mixing gal 4 (1 μmol/L) with different concentrations of D-(+)galactose (Sigma-Aldrich #48260–100G-F) or D(−)fructose (Sigma-Aldrich #47739; 50, 25, 12.5, or 0 mmol/L) and injecting the mixture over immobilized human CDε/δ under similar conditions as described above. PNGase F (Promega # V4831) treatment of human CDε/δ was performed in the Biacoret100 (GE healthcare/Cytiva) using a flow of 4 U/100 μL at a flow rate of 10 μL/min, with PNGase F in PBS over the immobilized CD3ε/δ (9,800 RU) for 600 seconds at 37°C. Galectin-4 (1 μmol/L) binding to the PNGase F-treated CD3ε/δ was performed as mentioned above. Enzymatic digestion was performed twice, and no change in binding was observed after the second digestion.

### Protein sequence alignments

CD3 protein sequences were aligned using MAFFT (39). The aligned sequences were trimmed, keeping the ectodomain corresponding to residues 23–100 of CD3-delta. Sequences used were human CD3D: NP_000723.1, human CD3G: NP_000064.1, mouse CD3D: NP_038515.3, mouse CD3G: NP_033980.1, sheep CD3D: NP_001009382.1, sheep CD3G: XP_004016118.2.

### Reanalysis of published data sets

Human data from Peng and colleagues (ref. 40; GSA: CRA001160), including 23 PDAC tumors and 12 nonpaired normal samples was reanalyzed to identify expression of *LGALS4* in various cell types. Using the author's annotation, 1,000 cells were randomly selected from each cluster with at most 100 cells per patient for each cluster. The raw UMI count data from the different patients were merged into a single object using Seurat version 4.1.0, and the data were normalized using the LogNormalize function. Normalized expression data from Boj and colleagues (31) was reanalyzed and visualized in GraphPad Prism for *Lgals4* RNA (TPM) and gal 4 protein (peak intensity) levels in normal PanIN and PDAC mouse organoids.

### Data and statistical analysis

Flowjo was used for flow cytometry data analysis. FMO was used to determine gate placement. Statistics were calculated using relative cell populations. Groups of animals in each experiment were determined by the number of animals required to gain sufficient statistical power. Power analysis was based on a pilot study performed in the lab and was approved by the animal ethics committee. Statistical analysis was performed in GraphPad Prism v10. Unpaired, nonparametric Mann–Whitney statistical tests were used for group comparisons unless otherwise indicated. Statistical significances are denoted as *, *P* < 0.05; **, *P* < 0.01; ***, *P*< 0.001.

### Data availability

Standard and publicly available tools were used as described previously. Code used to process the sequencing data and generate figures in R is available at Code Ocean (https://codeocean.com/capsule/3431369/tree/v1). Raw and processed sequencing data produced in the present study are available on the GEO with accession number GSE215389. Data are available on reasonable request from the corresponding author D.Ö.



## Results

### Gal 4 is elevated in the stroma of PDAC tumors

**Figure 1.**
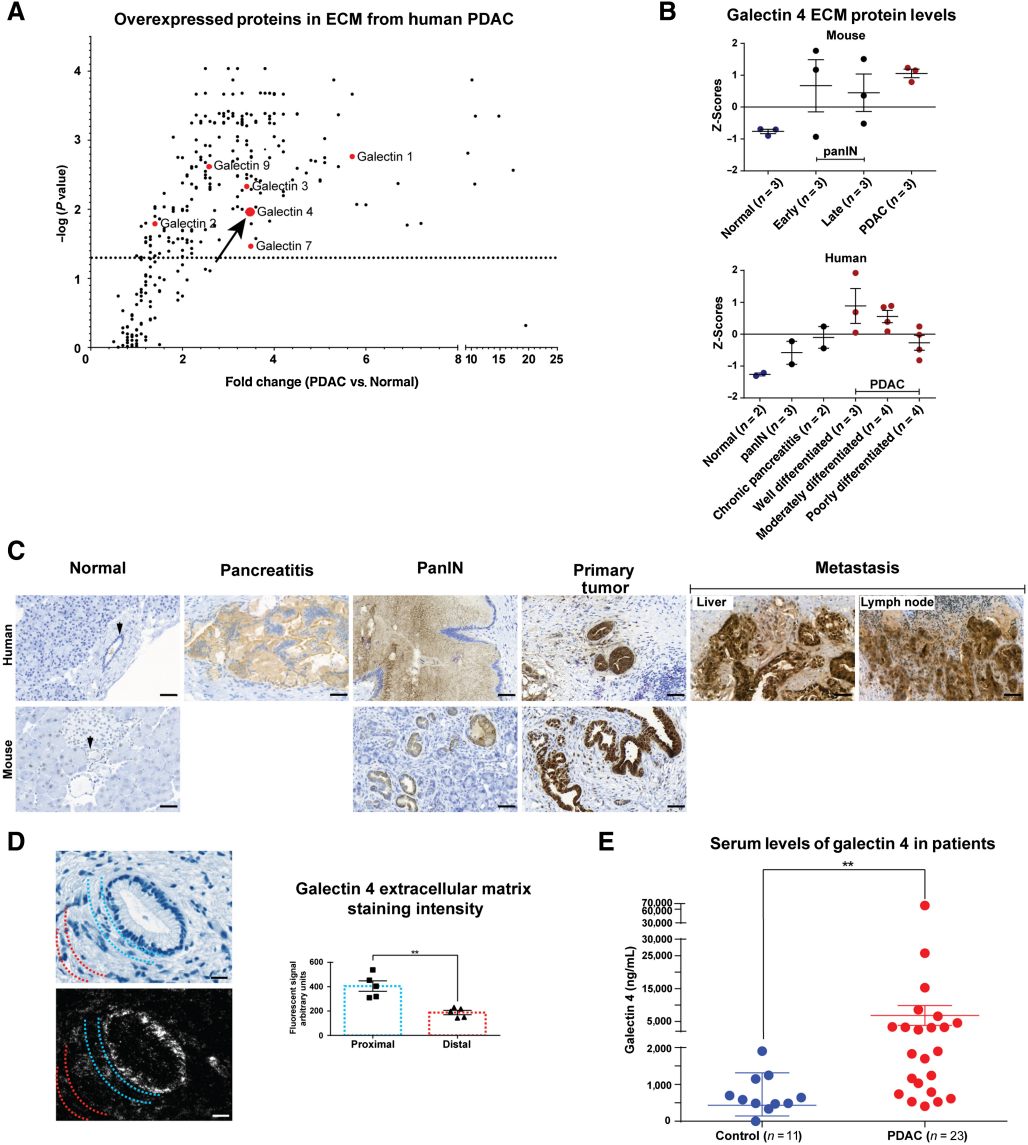
High levels of galectin 4 in pancreatic tumors and in the circulation of patients with pancreatic cancer. **A,** Volcano plot illustrating fold-change in extracellular expression of proteins detected from quantitative mass spectrometry performed on purified ECM from human PDAC tumors and normal pancreatic tissue (data from; ref. 22). Detected members of the galectin family of proteins in red. Arrow denotes galectin 4. Dotted horizontal line indicate significance level *P = 0.05* (*n* = 5 tumors, *n* = 2 normal pancreatic tissue). **B,** Mass spectrometry Z scores for galectin 4 (data from; ref. 22) in mouse and human tissue from normal pancreas, PanIN, chronic pancreatitis, and tumor samples (subcategorized by early- and late-stage for mouse samples). Error bars indicate mean ± SEM of Z-scores. **C,** Representative IHC staining of gal 4 in murine and human normal pancreatic tissue, human pancreatitis, murine and human PanIN precursor lesions, KPC-derived murine cancer and well-differentiated human PDAC, and human liver and lymph node metastases. Arrows denote normal pancreatic ducts. 5 samples from each tissue condition were stained; scale bar, 50 μm. **D,** PDAC tumors counterstained with HTX (top left) and labeled with UCNPs (bottom left) on the same section. Semiquantitative analysis of gal 4 signal intensity in areas proximal to or distal from the epithelial cells (right). The UCNP emission was quantified in areas proximal to epithelial cells (blue dotted lines represent the area where the luminescent signal was quantified) or distal from tumors (red dotted lines represent the area where the luminescent signal was quantified) in 5 tumors; scale bar, 20 μm. **E,** ELISAs to detect serum galectin 4 protein in patients with PDAC (*red*) and aged-matched controls (*blue*). Significance calculated using the Mann–Whitney test (*P* = 0.0012). Error bars show mean ± SEM. **, *P* < 0.01.

We examined galectins present in PDAC using a previously published and publicly available quantitative mass spectrometry data set generated from purified ECM (22). More than one hundred proteins with differential expression were detected when normal pancreatic tissue was compared with pancreatic tumor tissue in humans (Fig. 1A, reanalyzed from ref. 22). Various members of the galectin family showed significantly higher extracellular protein levels in PDAC tumors compared with normal pancreatic tissue (red dots in Fig. 1A). Publicly available transcriptomic data from the TCGA (26) further confirmed high expression of the galectin family of proteins in human PDAC tumors (Supplementary Fig. S1A). Most galectin family members exhibit immunomodulatory functions (11–13). Galectin 1 (41–43), 3 (44–46), and 9 (47–50) have been studied in PDAC, whereas the function of gal 4 is relatively unknown.

More detailed quantitative analysis of gal 4 in the mass spectrometry data set (22), comparing extracellular gal 4 protein levels between normal pancreatic tissue, chronic pancreatitis, PanIN (pancreatic intraepithelial neoplasia) precursor lesions, and PDAC showed increased gal 4 protein levels in the ECM with increased severity of lesions in both mouse and human tissue (Fig. 1B). Interestingly, the extracellular protein levels of gal 4 differ with different grades of tumor differentiation, where well and moderately differentiated PDAC tumors showed the highest gal 4 protein expression levels, gal 4 levels were comparatively lower in poorly differentiated tumors (Fig. 1B). Similar findings were reported by Hu and colleagues (21). Furthermore, levels of gal 4 were only slightly higher in the fibrotic stroma of patients with chronic pancreatitis, indicating elevated expression of gal 4 mainly when neoplastic cells are driving the desmoplastic process (Fig. 1B). IHC staining of tissues confirmed high abundance of gal 4 at the protein level in both mouse and human primary tumors, and human liver and lymph node metastases (Fig. 1C), whereas PanIN and chronic pancreatitis showed moderate expression of gal 4. To better visualize and quantify the extracellular deposits of gal 4, we used upconversion nanoparticles staining. Analysis of upconversion images of several tumors in a human patient with PDAC made it possible to detect and quantify gal 4 secreted to the stroma. These data indicated that gal 4 was secreted from tumors and found in higher abundance in the adjacent ECM, whereas gal 4 abundance decreased in ECM more distal to tumors (Fig. 1D).

### Patients with PDAC have elevated circulating gal 4

Because gal 4 expression was high in PDAC tissues, we used ELISAs to measure gal 4 in sera from patients with PDAC. We discovered that high intratumoral gal 4 expression found at the protein level in the mass spectrometry data set (22) and IHC analysis, and at the transcriptome level in the TCGA data set, translated to significantly higher serum levels in patients with PDAC compared with controls without malignant disease (Fig. 1E). We also found that control patients suffering from inflammatory conditions in the GI tract, with symptoms related to gastritis and IBDs, also showed high serum gal 4 (Supplementary Fig. S1B). These patients were therefore excluded from the analysis in Fig. 1E.

### Gal 4 overexpression is limited to carcinomas in the GI tract

Because gal 4 under normal physiological conditions is expressed in the GI tract but not in most other tissues (15), the expression of gal 4 was further analyzed in other cancer types from the TCGA. Indeed, we found similarly high gal 4 expression in several different GI cancers (Supplementary Fig. S1C), but low expression in respiratory, urinary, and genital cancers, indicating that gal 4 overexpression was limited to GI carcinomas. Next, we investigated the frequency of *LGALS4* (the gene encoding gal 4) alterations in GI, urinary, genital, respiratory, and breast carcinoma TCGA data sets. In pancreatic cancer, we found that *LGALS4* was amplified in 8% to 18% of sequenced tumors (Supplementary Fig. S1D), which in part explains the increased protein expression of gal 4 in PDAC.

### Epithelial cells are the cellular source of gal 4

**Figure 2.**
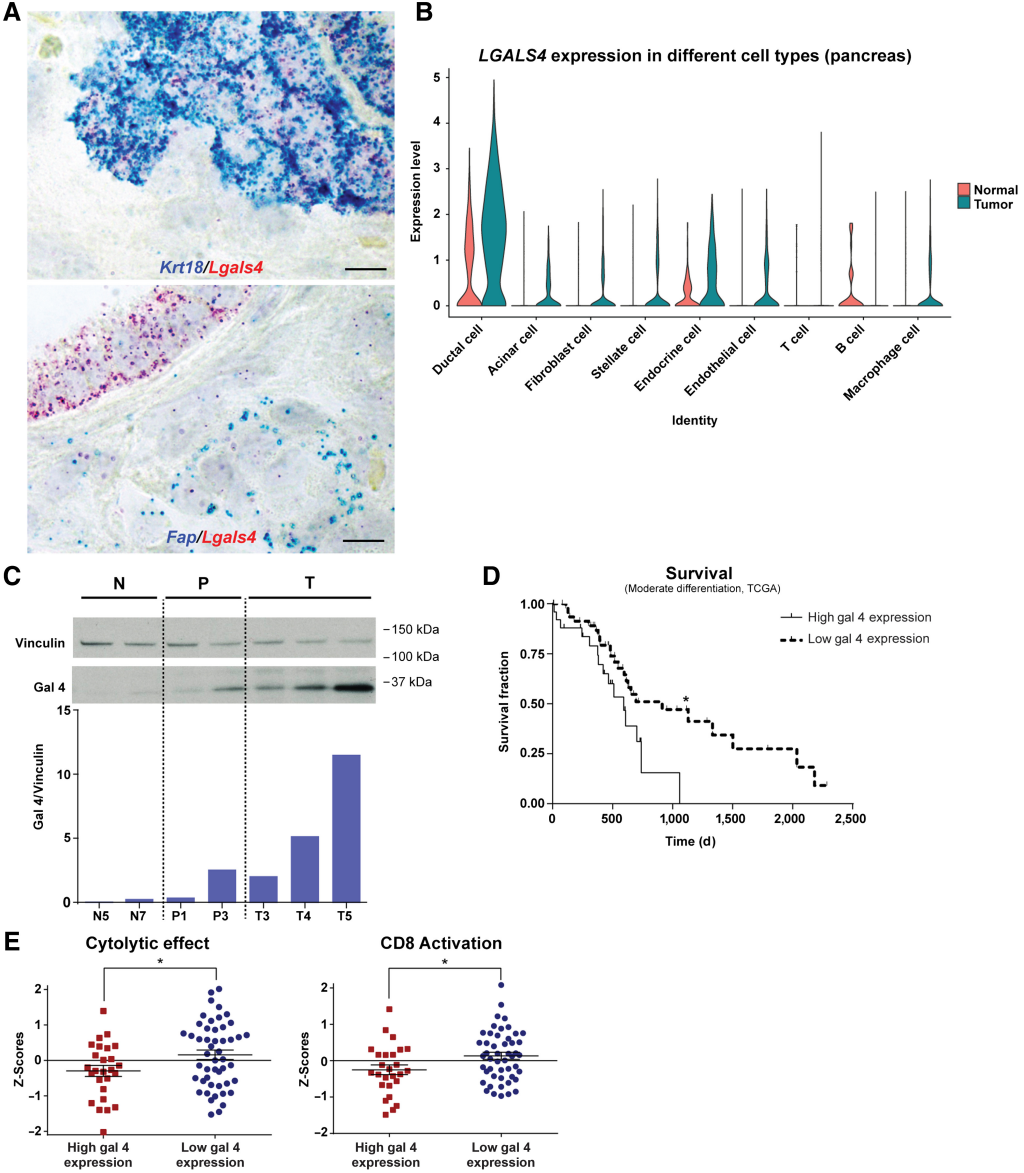
Galectin 4 is a cancer cell–derived marker of disease progression and prognosis in pancreatic cancer, with an association to reduced immune activity. **A,** Representative ISH images of cytokeratin 18 (*Krt18*, blue, top image) and fibroblast activation protein alpha (*Fap*, blue, the bottom image), together with gal 4 (*Lgals4*, in red), in mouse model of PDAC tumor tissue. 4 tumors analyzed in total; scale bar, 10 μm. **B,** Violin plot of single-cell RNA-seq data from Peng et al. (40) showing *LGALS4* expression in cell types from human PDAC tumors. **C,** Western blot and quantification (semiquantitative) of gal 4 protein in normal murine ductal organoid (N, two different cell lines), PanIN organoid (P, two different cell lines), and tumor organoid (T, three different cell lines) cell lines. Vinculin was used as loading control. **D,** Kaplan–Meier survival curve adapted from the TCGA data set (26), with patients grouped into high (*n =* 26) and low (*n =* 49) gal 4 mRNA expression (groups defined in Materials and Methods) and relationship to survival in patients with PDAC with moderately differentiated tumors. *, Significance (*P* = 0.0254) calculated using the Mantel–Cox test. **E,** Z-score comparison of high (*n =* 26) and low (*n = 49*) gal 4-expressing tumors within the group of moderately differentiated PDAC tumors in the TCGA data set. Genes associated with cytolytic effect of immune cells analyzed in the left graph (*P* = 0.036), and CD8 activation scores analyzed in the right graph (*P* = 0.0253). Genes used for CD8 activation and cytolytic effect are listed in Supplementary Table S2. *, Indicates significance calculated using an unpaired *t* test. Error bar indicates mean ± SEM.

To investigate the source of gal 4 found in the ECM of mouse PDAC tumors, we performed ISH to detect the cellular origin of gal 4 expression (Fig. 2A). The results showed colocalization of gal 4 transcripts (*Lgals4*) with the epithelial cell marker keratin 18 (*Krt18*), but not with the fibroblast marker fibroblast activation protein alpha (*Fap*), indicating epithelial cancer cells as the cellular origin of gal 4. In addition, analysis of a pancreatic cancer single-cell RNA-seq data set published by Peng and colleagues (40) confirmed that epithelial cells had the highest *LGALS4* mRNA expression, and that the expression was higher in cancer ductal cells compared with normal ductal cells (Fig. 2B).

### The expression profile of gal 4 is recapitulated in an organoid model of PDAC

Because epithelial cancer cells were the source of gal 4, we established murine pancreatic epithelial ductal organoid lines from the KPC mouse model with lesions from different stages of the disease. To confirm that these epithelial ductal organoids mirrored the gal 4 expression pattern observed *in vivo*, we performed Western blot analysis of organoid lines established from normal pancreatic tissue, tissue with PanIN lesions, and invasive carcinoma (Fig. 2C) and could detect a similar increasing gal 4 expression with severity of the lesion. This observation was further validated by reanalysis of previously published proteomic and transcriptomic data from murine pancreatic organoids by Boj and colleagues (ref. 31; Supplementary Fig. S1E). Furthermore, tumor organoids isolated from metastatic lesions in KPC mice expressed higher gal 4 compared with organoids established from primary tumors (Supplementary Fig. S1F), indicating that the high expression of gal 4 was maintained at the metastatic site, which was also apparent in human liver and lymph node metastases (Fig. 1C).

### High gal 4 is associated with worse survival and reduced immune activity

We next investigated the association between *LGALS4* expression and survival using the TCGA data set. When all patients were included in the analysis, no significant difference in survival between patients with PDAC with low and high expression of gal 4 was observed (Supplementary Fig. S1G). To separate the cell-intrinsic effect of gal 4 on cancer cell differentiation from that of the secreted extracellular effect, we grouped patients based on differentiation grade. With this approach, we observed that high gal 4 expression negatively associated with survival in moderately differentiated PDAC (Fig. 2D).

Previous studies have indicated the role of gal 4 in T-cell modulation (15). To delineate if gal 4 expression in PDAC correlated to immune cell activity, we investigated the cytolytic effect, the differential expression of genes related to cytotoxic potential, and CD8 activity score (listed in Supplementary Table S2, from Azizi and colleagues; ref. 29), within the group of patients with moderately differentiated tumors. A significant association between the cytolytic effect and CD8 activation score in the group of patients with low expression of gal 4 (Fig. 2E), implying an immunosuppressive role of gal 4 in PDAC. To verify if this correlation between immune cell activity and gal 4 expression also occurred in other GI cancers, analysis of the TCGA colorectal cancer data set was performed. Like in PDAC, cytolytic effect and CD8 activity was higher in patients with colorectal cancer with low gal 4 expression (Supplementary Fig. S1H). We also discovered that gal 4 expression inversely correlated to the checkpoint inhibitor target PDL1 in PDAC (Supplementary Fig. S1I), indicating that in each individual case, different immunosuppressive factors might be the dominant contributor to the immunosuppression.

### Gal 4 regulates immune cell infiltration in a murine model of PDAC

The gal 4 expression profile in PDAC, the elevated gal 4 serum levels in patients with PDAC, and the association between gal 4 expression, survival, and cytolytic effect prompted us to perform additional investigations. With CRISPR/Cas9 methods, we knocked-out gal 4 in murine KPC tumor-derived cell lines using guide RNAs (sgRNA) directed against the gal 4 locus (Gal 4–KO, Supplementary Fig. S2A) and the inert R26 locus as control (R26 control). Gal 4–KO status was verified with Western blots for the sgRNA directed against exon 2 of *Lgals4* (Supplementary Fig. S2B). Cell lines with gal 4–KO status did not differ in cell survival or proliferation rate *in vitro* compared with R26 controls (Supplementary Fig. S2C and S2D). This verified that gal 4 does not have an intrinsic effect on cell proliferation and survival *in vitro*. Sanger sequencing further verified alterations of the gal 4 gene at the intended targets, generating insertion/deletion and frameshifts (Supplementary Fig. S2E) predicted to yield no functional protein (Supplementary Fig. S2F).

**Figure 3.**
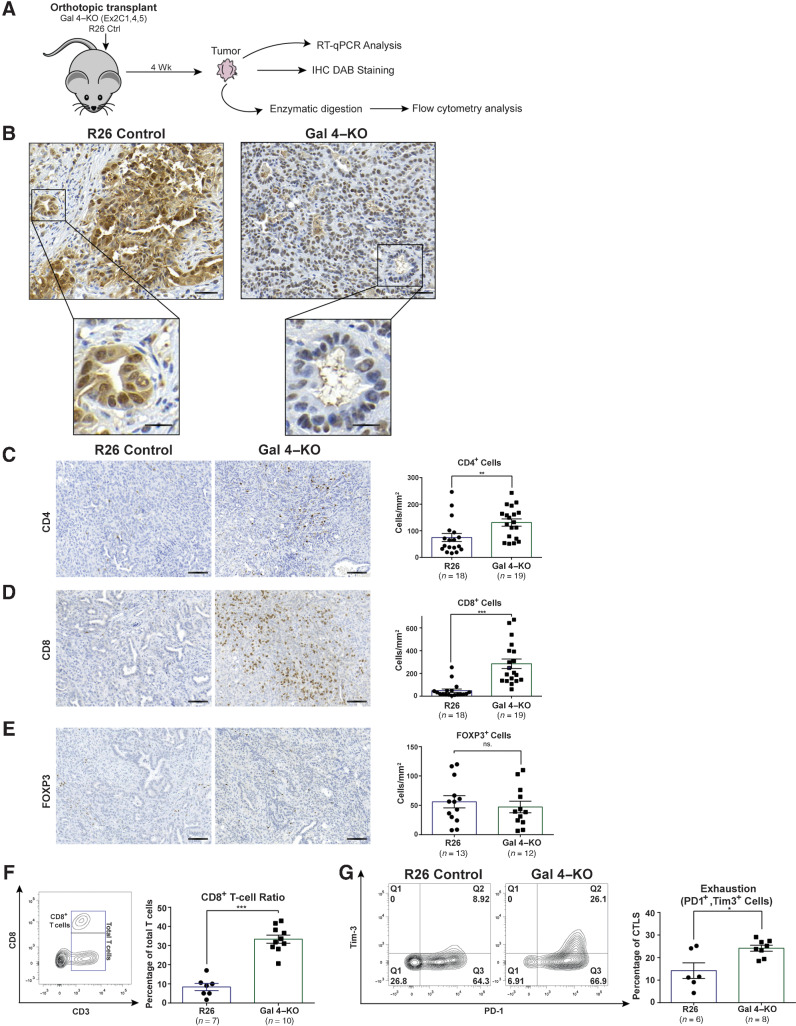
Orthotopic transplantation of gal 4–KO cell lines identifies differences in CD4^+^ and CD8^+^ cell infiltration and function. **A,** Schematic of the orthotopic transplantation experiments using gal 4–KO and R26 control cell lines transplanted into immune competent C57BL6/J mice. **B,** Representative IHC images of gal 4 in R26 control and gal 4–KO tumors (top scale bar, 50 μm; bottom, scale bar, 20 μm). A total of 10 tumors were stained, 5 of each condition. **C–E,** Representative IHC images (*left*) and quantification (*right*) of CD4^+^ cells (*P* = 0.0032; **C**), CD8^+^ cells (*P* = <0.0001; **D**), and FOXP3^+^ cells (ns., no significant difference; **E**), in orthotopically transplanted tumors with gal 4 (R26 controls) and no gal 4 expression (Gal 4–KO); scale bar, 100 μm. Significance calculated using the Mann–Whitney test. Error bars show mean ± SEM. **F,** Representative contour plots illustrating gating strategies of intratumoral CD8^+^ T cells (*left*). Scatter plot with bar graph showing the fraction of CD8^+^ cells out of the total amount of CD45^+^ CD3^+^ cells in R26 control and gal 4–KO tumors (*right*; *P* = 0.0001). Significance calculated using the Mann–Whitney test. Error bars show mean±SEM. **G** Representative contour plots of intratumoral CD45^+^, CD3^+^, CD8^+^ cells (CD8^+^ T cells gated as in Fig. 3 F) expressing the exhaustion markers PD-1 (*x*-axis) and Tim-3 (*y*-axis) in R26 control and gal 4–KO tumors (*left*). Scatter plot with bar graph s (*right*) showing quantification of CD8^+^ T cells exhaustion (PD1^+^ and Tim-3 cells in Q2) in R26 control and gal 4–KO tumors (*P* = 0.046). Significance calculated using the Mann–Whitney test. Error bars show mean with SEM. **, *P* < 0.01; ***, *P* < 0.001.

To investigate the effect of gal 4 on immune cell infiltration, we performed orthotopic transplantations of gal 4–KO and R26 control cells into the splenic lobe of the pancreas of immune competent *C57/BL6J* mice (Fig. 3A). We found no significant difference in tumor weight at the endpoint (4 weeks) between R26 control and gal 4KO tumors (Supplementary Fig. S2G). IHC analysis of tumor tissues at the endpoint confirmed gal 4–KO status in the tumors (Fig. 3B). We proceeded to systematically quantify the subtypes of T cells infiltrating the tumors by performing IHC for CD4^+^, CD8^+^, and FOXP3^+^ cells to identify T-helper cells, cytotoxic T cells (CTL), and regulatory T cells (Treg), respectively (Fig. 3C–E). The analysis demonstrated a significant increase in CD4^+^ and CD8^+^ T cells in gal 4–KO tumors compared with R26 control tumors (Fig. 3C and D), but no difference in the number of infiltrating FOXP3^+^ Tregs (Fig. 3E).

### Intratumoral T cells in gal 4–KO tumors show a high CD8^+^ T-cell ratio and exhaustion

The significantly higher number of CD8^+^ cells in the gal 4–KO tumors suggested a direct effect of gal 4 on antitumor effector cells. To evaluate whether gal 4 affected the function of infiltrating immune cells, we performed flow cytometry analysis of intratumoral immune cells from R26 control and gal 4–KO tumors (Fig. 3A). Infiltrating CD8^+^ T cells (CD45^+^CD3^+^CD8^+^) normalized to total T-cell infiltration (Supplementary Fig. S2H) was significantly higher in gal 4–KO tumors compared with R26 controls (Fig. 3F). This was consistent with our IHC findings (Fig. 3D) and defined the infiltrating CD8^+^ cells as CD8^+^ T cells. However, we found no difference in CD8^+^ T-cell activation or degranulation in gal 4–KO tumors compared with controls (Supplementary Fig. S2I). In contrast, we found that infiltrating CD8^+^ T cells in gal 4–KO tumors had significantly higher expression of the exhaustion markers PD-1 and Tim-3 compared with immune cells infiltrating R26 control tumors (Fig. 3G), indicating reduced antitumor responses in gal 4–KO tumors. To verify a functional difference in the exhausted CD8^+^ T cells, we performed RT-qPCR analysis of R26 control and gal 4–KO tumors and found a trend toward higher *Tnf* expression and significantly higher expression of *Nfkb1* (Supplementary Fig. S2J), suggesting a higher rate of inflammation in R26 control tumors compared with gal 4–KO tumors, consistent with the higher rate of CD8^+^ T-cell exhaustion in these tumors.

### Reduced gal 4 expression results in increased survival in an organoid-based, orthotopic transplantation model

To create a model system that more accurately mimicked human disease, we developed a transplantation model using slower-growing KPC tumor organoids with a constitutively expressed hairpin directed against gal 4, generating gal 4-knockdown organoids (Gal 4–KD), using a scramble hairpin sequence as the negative control (scramble). First, we evaluated 5 different hairpins against gal 4 and selected the 2 hairpins with the highest knockdown efficacy in a KPC 2D cell line (Supplementary Fig. S3A). The selected hairpins were used to generate four KPC tumor organoid lines with stable expression of gal 4 hairpins. These cells exhibited significantly reduced, but not fully eliminated, gal 4 expression compared with scramble-hairpin controls (Supplementary Fig. S3B). We verified that gal 4 did not have a cell intrinsic effect on proliferation of the organoids by comparing proliferative rates following gal 4–knockdown (Supplementary Fig. S3C). The cell-intrinsic effect of gal 4 has been suggested to increase the degree of differentiation of cancer cells and reduce EMT via downregulation of Wnt signaling (19). This led us to evaluate changes in EMT markers in the generated organoid lines, and indeed we noted that gal 4–KD organoids showed elevated expression of genes common for EMT (Supplementary Fig. S3D).

**Figure 4.**
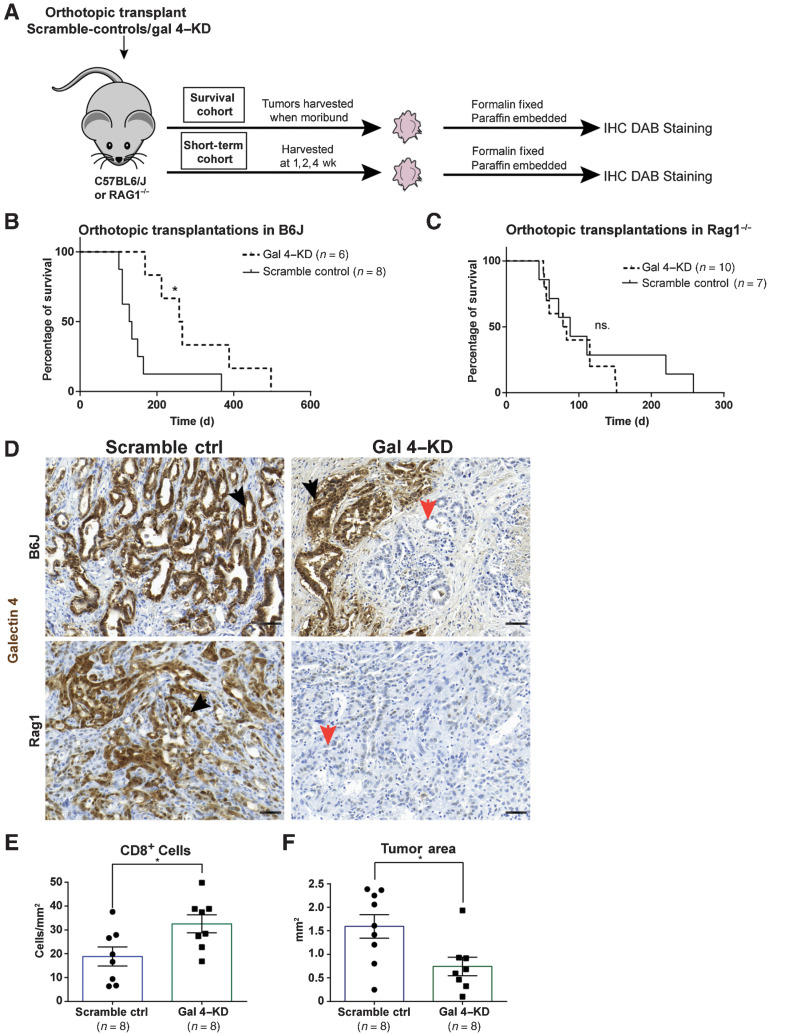
Reduced galectin 4 expression is linked to better survival in an organoid-based PDAC transplantation mouse model. **A,** Schematic of orthotopic transplantation of Gal-4KD and scramble-control organoid lines into *C57BL6/J* and *RAG1^−/−^* mice. Tumors harvested at time of death (survival cohort), or at 1, 2, and 4 weeks timepoints (short-term cohort), followed by IHC analysis of tumor tissue. **B** and **C,** Kaplan–Meier Survival curve of orthotopic transplantations of gal 4–KD and scramble-control organoid lines into *C57BL6/J* mice (**B**) and *RAG1^−/−^* mice (**C**). Statistical significance (*) calculated by the Mantel–Cox test (*P* = 0.014; ns., no statistical difference). **D,** Representative IHC DAB staining of gal 4 in scramble-control and gal 4–KD tumors in *C57BL6/J* and RAG1^−/−^ mice. Black arrows denote gal 4-positive and orange arrows denote negative tumors (scale bar, 50 μm). **E** and **F,** Scatter plot with bar graphs showing quantification of CD8^+^ cells/mm^2^ (**E**) and tumor area mm^2^ (**F**) in scramble-control and gal 4–KD tumors up to the 2-week timepoint (including 7- and 14-day tumors) of short-term orthotopic transplants. Statistical significance (*) calculated using the Mann–Whitney test (for CD8, *P* = 0.028, tumor area *P* = 0.027). Error bars show mean ± SEM.

Tumor organoids with verified reduced gal 4 expression (Gal 4–KD) and scramble-hairpin controls were orthotopically transplanted into immunocompetent C57/BL6J mice to investigate the effect of gal 4 on survival (Fig. 4A). A significant difference in survival was observed between mice implanted with gal 4–KD organoids compared with scramble-control organoids (Fig. 4B), which was not observed when the same organoid lines were transplanted into immunocompromised RAG1^−/−^ mice lacking mature B and T cells (Fig. 4C). Verification of gal 4 status at the experimental endpoint in the C57/BL6J mice showed a recurrence of gal 4 expression in gal 4–KD–transplanted mice but was not observed in the immunocompromised RAG1^−/−^ mice (Fig. 4D). IHC analysis of CD4^+^ and CD8^+^ cell infiltration into the tumor cores at the survival endpoint (Supplementary Fig. S3E) did not differ between scramble-control and gal 4–KD organoids. Similarly, we could not detect any difference in tumor weight between controls and gal 4–KD tumors at the endpoint (Supplementary Fig. S3F).

### Reduced gal 4 expression results in increased infiltration of CD8^+^ cells and reduced tumor area

The immune-mediated differences in survival and apparent recurrence in gal 4 expression prompted additional investigation of the immune response immediately after transplantation. We therefore proceeded with short-term transplants (ranging between 7 and 28 days) of scramble-control and gal 4–KD organoid lines (Fig. 4A). IHC staining of CD8^+^ cells and epithelial cell apoptosis (cleaved caspase 3) 1 to 2 weeks after transplantation of gal 4–KD and scramble-control cell lines revealed higher infiltration of CD8^+^ cells per mm^2^ tumor (Fig. 4E), but no significant difference in apoptotic epithelial cells (Supplementary Fig. S3G). We additionally evaluated the tumor area of gal 4–KD and scramble-control tumors up to 14 days after transplantation and found that gal 4–KD tumors had significantly smaller tumor areas compared with scramble controls (Fig. 4F), and the gal 4 recurrence observed in the survival experiment (Fig. 4D) was also observed in short-term transplants (Supplementary Fig. S3H). Together these data demonstrate that the immune cell-mediated effect on tumor cells in gal 4–KD transplants occurs shortly after transplantation and has already taken place before the 1-week time point. The details of this process could not be further studied in the transplantation model because no organized tumors could be identified and processed earlier than 1 week after transplantation.

### Gal 4 induces changes in the myeloid cell compartment

**Figure 5.**
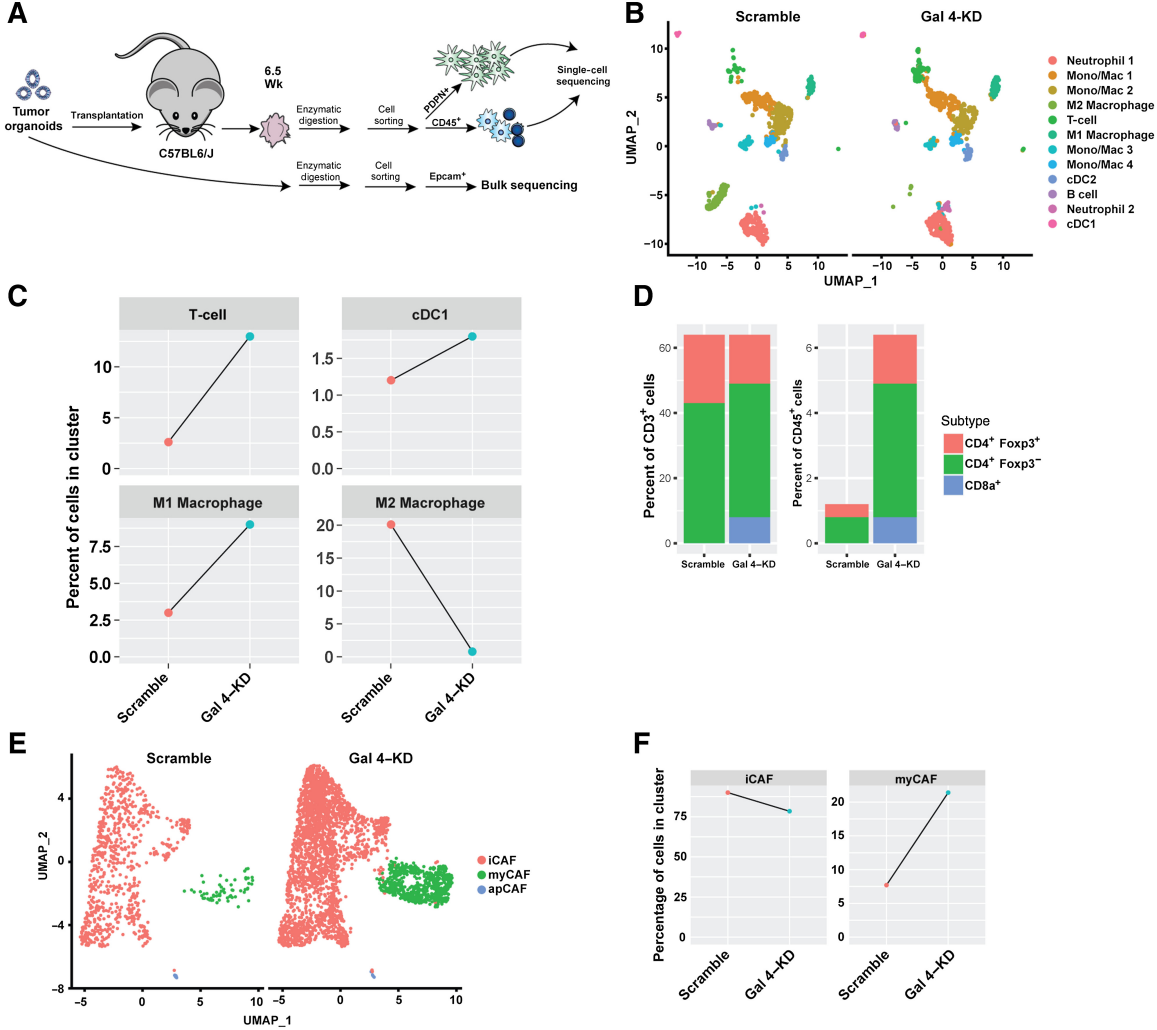
Single-cell RNA-seq of scramble-control and gal 4–KD orthotopic transplants, with a focus on immune cell subpopulations and CAF subtypes. **A,** Schematic of orthotopic transplantation of gal 4–KD and scramble-control organoid lines into *C57BL6/J* mice. 4 tumors were harvested at 6.5 weeks, followed by dissociation, cell sorting of podoplanin^+^ and CD45^+^ cells and 2 were selected for 10x single-cell sequencing based on the sample quality. A total of 7,677 cells were analyzed. Epcam^+^ cells from gal 4–KD and scramble-control organoid lines bulk-sequenced using Smart-Seq2. **B,** Uniform Manifold Approximation and Projection (UMAP) plot showing all detected immune cell (CD45^+^) populations from single-cell sequencing of scramble-control and gal 4–KD tumors in orthotopic transplants. Mac, macrophage; Mono, monocytes; cDC, conventional DC subsets. **C,** Line graph showing differences in the percentage of cells in clusters of T cells, M1 macrophages, M2 macrophages, and DCs between scramble-control and gal 4–KD orthotopic transplants. **D,** Bar graph showing differences in ratio of CD8a^+^, CD4^+^Foxp3^−^, and CD4^+^FOXP3^+^ cells in all CD3^+^ cells or all CD45^+^ cells. **E,** UMAP plot showing all detected CAF populations from single-cell sequencing of scramble-control and gal 4–KD tumors in orthotopic transplants. **F,** Line graph showing differences in the percentage of cells in clusters of iCAFs and myCAFs between scramble-control and gal 4–KD orthotopic transplants.

To investigate changes in myeloid cell populations, we performed single-cell RNA-seq on orthotopic scramble and gal 4–KD organoid transplants from C57BL6/J mice, and bulk sequencing of cell lines before transplantation to verify gal 4–KD status (Fig. 5A). Bulk sequencing of EPCAM^+^ epithelial cells showed downregulation of *Lgals4* in cell lines before transplantation (Supplementary Fig. S4A). In the single-cell data, we identified most immune cell types in distinct clusters within intratumoral CD45^+^ cells (Fig. 5B, Supplementary File S2), and differences in immune cell clusters between scramble-control and gal 4–KD tumors were observed (Fig. 5C, Supplementary Fig. S4B). The proportion of M1 macrophages, T cells, and antigen-presenting dendritic cells, known to be prognostically favorable in PDAC (51), was increased in gal 4–KD tumors (Fig. 5C). In contrast, the proportion of immunosuppressive M2 macrophages was downregulated in the gal 4–KD transplants (Fig. 5C, Supplementary Fig. S4C). We additionally found that the ratio of CD8^+^ T cells normalized to total T cells or total CD45^+^ cells was higher in gal 4–KD tumors (Fig. 5D), confirming our results from the gal 4–KO transplantation model (Fig. 3F). T cells in the gal 4–KD tumors also showed higher expression of activation markers and cytotoxicity genes (Supplementary Fig. S4D–S4F), in accordance with the results from the analysis of the TCGA data sets (Fig. 2E). Altogether, the sequencing data showed that reducing the gal 4 expression the immune microenvironment leads to a general increase in antitumor activity, with increased T-cell activation, M1 macrophage polarization, reduced proportion of immunosuppressive cells, and increased fraction of antigen-presenting cells.

### Gal 4 induces changes in fibroblast subtypes

Furthermore, we investigated changes in cancer-associated fibroblast (CAF) subtypes in scramble-control and gal 4–KD tumors by performing single-cell sequencing on flow cytometry–sorted podoplanin^+^ (PDPN^+^) cells (as depicted in Fig. 5A). We identified the most common CAF subtypes, including myofibroblastic CAFs (myCAF), inflammatory CAFs (iCAF), and mesothelial antigen-presenting CAFs (apCAF; Fig. 5E; Supplementary File S3). Gal 4–KD tumors showed a higher proportion of myCAFs and lower proportion of iCAFs compared with scramble-control tumors (Fig. 5F). We also found significantly upregulated myCAF markers in gal 4–KD tumor CAFs, including *Col12a1*, *Col8a1*, *Thbs2*, and *Igfbp3* (Supplementary Fig. S4G). In contrast, iCAF markers were significantly downregulated in the gal 4–KD tumors, including *Klf4*, *Ly6c*, and *Igfbp5* (Supplementary Fig. S4G). In addition, we found a trend, indicating that the apCAF subtype was more abundant in scramble controls (Supplementary Fig. S4H), although the number of apCAF cells were too few to make any robust conclusions. However, apCAFs in scramble tumors showed higher expression of mesothelial markers and similar expression of MHC II pathway genes (Supplementary Fig. S4I–S4J). Mesothelial genes in apCAFs are previously described to decrease when mesothelial cells transform into apCAFs (40). Together, our results showed differences in myCAF, iCAF, and apCAF subtypes between gal 4-producing scramble-control and gal 4–KD tumors, indicating that reduced gal 4 expression associates with less inflammatory and more myofibroblastic CAF subtypes.

### Gal 4 induces T-cell apoptosis through interaction with CD3

**Figure 6.**
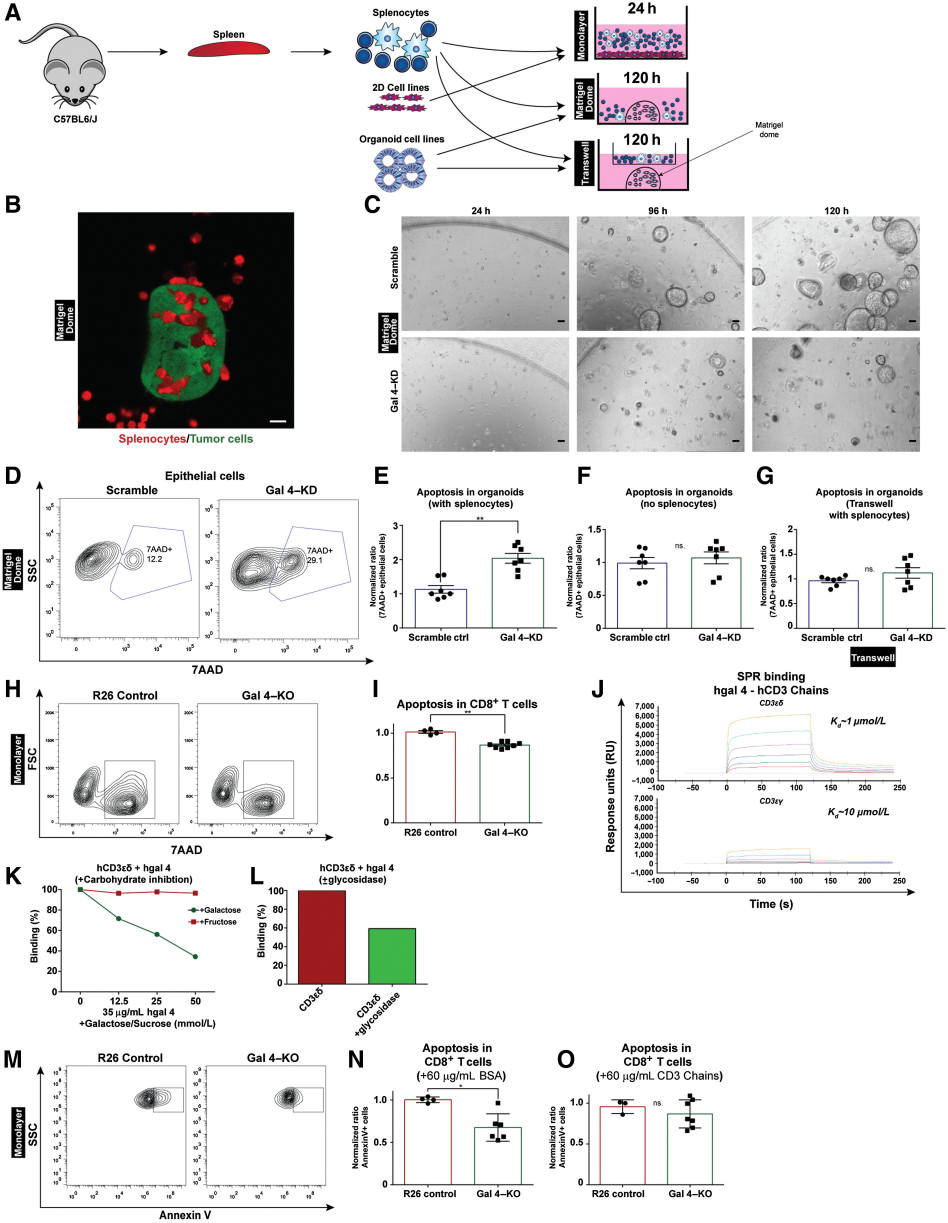
*Ex vivo* models of PDAC reveal immunosuppressive effects of galectin 4 through CD3δ. **A,** Schematic depicting two *ex vivo* model systems using splenocytes isolated from immunocompetent C57BL6/J mice added to KPC-derived 2D cells (*Monolayer model*) or tumor organoids/tumor spheroids (*Matrigel dome and Transwell models*). Splenocytes were isolated from aged-matched C57BL6/J mice. **B,** Representative confocal microscope image of a KPC cell–derived spheroid (GPF, green) surrounded by T cells (cell tracker deep red stain, red) after 48 hours of incubation; scale bar, 10 μm. **C,** Representative brightfield images of gal 4–KD and scramble-hairpin control organoids at 24, 96, and 120 hours in cocultures with immune cells (scale bar, 100 μm). The experiment was repeated 3 times. **D,** Representative contour plot showing gating strategy for 7AAD^+^ epithelial cells from dissociated Matrigel domes in scramble-hairpin controls and gal 4–KD cell lines in the tumor organoid *ex vivo* model. Quantifications from this experiment are presented in **E–G**. **E–G,** Scatter plot with bar graph showing flow cytometry quantification of apoptosis in organoid cells from dissociated Matrigel domes with added splenocytes (**E**), without splenocytes (organoids only; **F**), organoids with splenocytes in Transwell at 120 hours timepoint (**G**). Scramble-hairpin controls (*n = 6*) and gal 4–KD (*n = 7*); ns., not significant. **H,** Representative contour plots showing gating strategy for 7AAD^+^CD8^+^ T cells from the monolayer model using R26 control and gal 4–KO cell lines. Quantifications from this experiment are presented in **I**. **I,** Scatter plot with bar graph showing flow cytometry quantification of 7AAD^+^CD8^+^ T cells added to R26 control (*n* = 4) and gal 4–KO (*n* = 8) in the monolayer model (R26 normalized to 1) at 24 hours in culture. Significance (**, *P* = 0.004) calculated using the Mann–Whitney test. **J,** SPR binding graph showing binding between human recombinant gal 4 (hgal 4) and human recombinant CD3ε/δ and CD3ε/γ heterodimer chains. The experiment was repeated 3 times. **K,** Line graph of SPR data for carbohydrate inhibition of binding between human recombinant gal 4 and human recombinant CD3ε/δ heterodimer treated with either galactose or fructose. The experiment was repeated once for fructose and twice for galactose. **L,** Bar graph of SPR data for binding between human recombinant gal 4 and human recombinant CD3ε/δ heterodimer following treatment with a glycosidase targeting N-linked glycosylations. The experiment was repeated 2 times. **M,** Representative contour plot showing gating strategy for AnnexinV^+^CD8^+^ T cells from monolayer model used in the gal 4-blocking assay. Quantifications from this experiment are presented in **N–O**. **N,** Scatter plot with bar graph showing flow cytometry quantification of AnnexinV^+^CD8^+^ T cells added to R26 control (*n* = 4) and gal 4–KO (*n* = 6) supplemented with BSA in the monolayer model (R26 normalized to 1). Significance (*P* = 0.019) calculated using the Mann–Whitney test. **O,** Scatter plot with bar graph showing flow cytometry quantification of AnnexinV^+^ CD8^+^ T cells added to R26 control (*n* = 3) and gal 4–KO (*n* = 7) supplemented with 60 μg/mL soluble CD3 chains in the 2D cell-based *ex vivo* model (R26 normalized to 1). Non-significant, ns.; *, *P* = 0.5167 calculated using the Mann–Whitney test.

To investigate the mechanisms behind the differences in infiltration of CD8^+^ cells in tumors and the difference in survival seen in the transplantation experiments (Figs. 3 and 4), we developed *ex vivo* model systems in which immune cells from C57BL6/J murine spleens were co-incubated with KPC 2D cell lines to determine the effect of extracellular gal 4 on T cells, or co-incubated with gal 4-producing organoids to determine the effect on epithelial cell apoptosis (Fig. 6A). Brightfield image analysis of the organoid cocultures revealed that lymphocytes were able to infiltrate Matrigel domes containing cancer cells (Supplementary Fig. S5A). Using GFP-expressing cancer cells and T cells labeled red, we visualized T cells in direct contact with cancer cells after 48 hours (Fig. 6B). Image analysis of the organoid/immune cell cocultures showed a clear difference in organoid growth and survival between scramble-control and gal 4–KD cells, where cancer cells expressing gal 4 (scramble controls) formed larger organoids after 120 hours (Fig. 6C, *top*), whereas gal 4–KD cancer cells formed smaller and fewer organoids (Fig. 6C, *bottom*) when cocultured with immune cells.

To study the cause of this difference in organoid growth and survival, control and knockdown cells were analyzed using flow cytometry (Fig. 6D–G; Supplementary Fig. S5B). We found a significant increase in apoptotic (7AAD^+^) epithelial cells in gal 4–KD organoids compared with scramble controls when exposed to splenocytes (Fig. 6E). Without splenocytes, we did not find any significant difference in apoptosis between the cell lines (Fig. 6F), or when cultured together with immune cells in a Transwell inserts without direct contact between the two different cell types (Fig. 6G). These data indicate that direct interaction between cancer cells and splenocytes are needed for the observed phenotype.

To evaluate the effect of secreted gal 4 on immune cells, we measured apoptosis in T cells cultured with gal 4–KO and R26 control 2D KPC cell lines (Supplementary Fig. S5C) and analyzed CD8^+^ T cells using flow cytometry (Fig. 6H). A rapid induction of apoptosis in CD8^+^ T cells was seen when co-incubated with the gal 4–producing R26 control cell line (Fig. 6I). It has previously been described that gal 4 can bind to CD3 (15). To further characterize the binding between CD3 and gal 4, we performed a series of SPR-binding experiments (Fig. 6J–L). CD3 consists of three distinct protein chains, epsilon (ε), gamma (γ), and delta (δ), forming cell surface heterodimers with intracellular signaling complexes, ITAMs. CD3 heterodimers together with the αβ chains of the T-cell receptor (TCR) make up the TCR complex (52). Biochemical and structural studies have revealed N-glycosylation sites in CD3γ and CD3δ chains (53–55), and glycosylation of human CD3δ, but not CD3γ, are important for TCR complex assembly (55). Alignments of CD3 protein sequences showed that N-linked glycosylation residues in the delta chain were highly conserved between species (Supplementary Fig. S5D). Using CD3δ/ε heterodimers and recombinant human gal 4 (hgal 4), we detected binding for both human and mouse CD3δ/ε chains (Fig. 6J; Supplementary Fig. S5E). The affinity to CD3δ/ε was 10-times higher than to CD3γ/ε (Fig. 6J). Furthermore, this binding could be blocked using galactose, whereas a fructose control did not block the binding (Fig. 6K), indicating that hgal 4 specifically binds the precursor to glycosylation, galactose. Enzymatic treatment with a glycosidase that cleaves N-linked glycosylations reduced the binding between hgal 4 and the CD3δ/ε heterodimer by approximately 50%, indicating that this binding is, at least in part, glycosylation-dependent (Fig. 6L). To evaluate the effect of CD3 chains in a biological system, we added soluble CD3 chains to gal 4 producing R26 control and gal 4–KO cell lines when cultured with pre-activated CD8^+^ T cells. We analyzed the proportion of Annexin V^+^ cells using flow cytometry (Fig. 6M; Supplementary Fig. S5F). As before, we found significantly higher apoptosis in CD8^+^ T cells when cultured with gal 4-producing R26 controls compared with when cultured with gal 4–KO cells (Fig. 6N). Addition of soluble CD3 chains removed this difference (Fig. 6O), indicating that CD3 competitively binds gal 4 in solution. In summary, these experiments showed that gal 4 interacts with CD3 on T cells and induces apoptosis.

## Discussion

PDAC cells exhibit multiple mechanisms to induce immunosuppression and achieve immune evasion. Developing treatments to overcome immunosuppression could reduce tumor burden and increase survival in patients with PDAC. Previous studies have identified galectin family members 1 (41–43), 3 (44–46), and 9 (48, 49) as immunosuppressive proteins in PDAC. In this study, we identified gal 4 as an important immunomodulatory factor in PDAC and found that gal 4 was expressed at high levels in cancer cells and secreted to the tumor stroma, where it induced apoptosis in infiltrating T cells. Gal 4 is normally expressed in the GI tract and is reported to have distinct cell-intrinsic and extracellular effects. Extracellular gal 4 is involved in immune modulation (18) and T-cell apoptosis (15) in intestinal inflammation. This immunosuppressive role, together with the expression pattern throughout the GI tract, indicates that gal 4 might be involved in immune tolerance toward food antigens. Gal 4 has previously been of interest in inflammatory bowel diseases, where gal 4 exacerbates inflammation and delays healing of the inflamed colon (18). Gal 4 expression has also been investigated in ulcerative colitis without a clear link to disease severity (56).

We showed that gal 4 is overexpressed in the ECM of PDAC tumors compared with normal pancreatic tissue and that extracellular protein expression of gal 4 increased throughout the progression of the disease. With image analysis, we also detected gal 4 secretion, suggesting that secreted gal 4 gets trapped in the ECM, which was detected using mass spectrometry (20). Elevated gal 4 was also detected in the circulation, suggesting that gal 4 could be potentially used as a PDAC biomarker for diagnosis and to monitor treatment response, or to identify patients with disease recurrence (also suggested by Hu and colleagues; ref. 21). We also found high gal 4 expression in several different GI cancers, and consequently, circulating gal 4 could potentially be used as a biomarker in diagnosis or monitoring of other GI cancers.

Our data also showed that inflammation in the GI tract could induce gal 4 expression, which is in contrast with the work by Yu and colleagues (57), where the authors could not detect a difference in gal 4 serum levels between patients with inflammatory bowel disease and healthy controls. However, the role of gal 4 in other diseases of the GI tract, and potentially inflammation, indicates that this will need to be taken into consideration if gal 4 is further developed into a screening tool for GI carcinomas.

Low intracellular gal 4 has been reported to contribute to reduced differentiation, increased migration, and metastasis in PDAC (19–21), features associated with EMT. The difference in gal 4 expression, we observed in well differentiated and poorly differentiated tumors could be explained by the difference in function between intracellular and extracellular gal 4. In our model systems, we also observed that reducing gal 4 expression–induced markers associated with EMT, which is in line with previously published studies on the cell intrinsic effect of gal 4 (19–21). Both high expression of gal 4, which induces immunosuppression, and low expression of gal 4, which stimulates EMT, motility, and infiltration, can be beneficial to tumor progression. Importantly, this highlights that a future therapeutic approach should only target extracellular gal 4 and not affect the intracellular role of gal 4.

**Figure 7.**
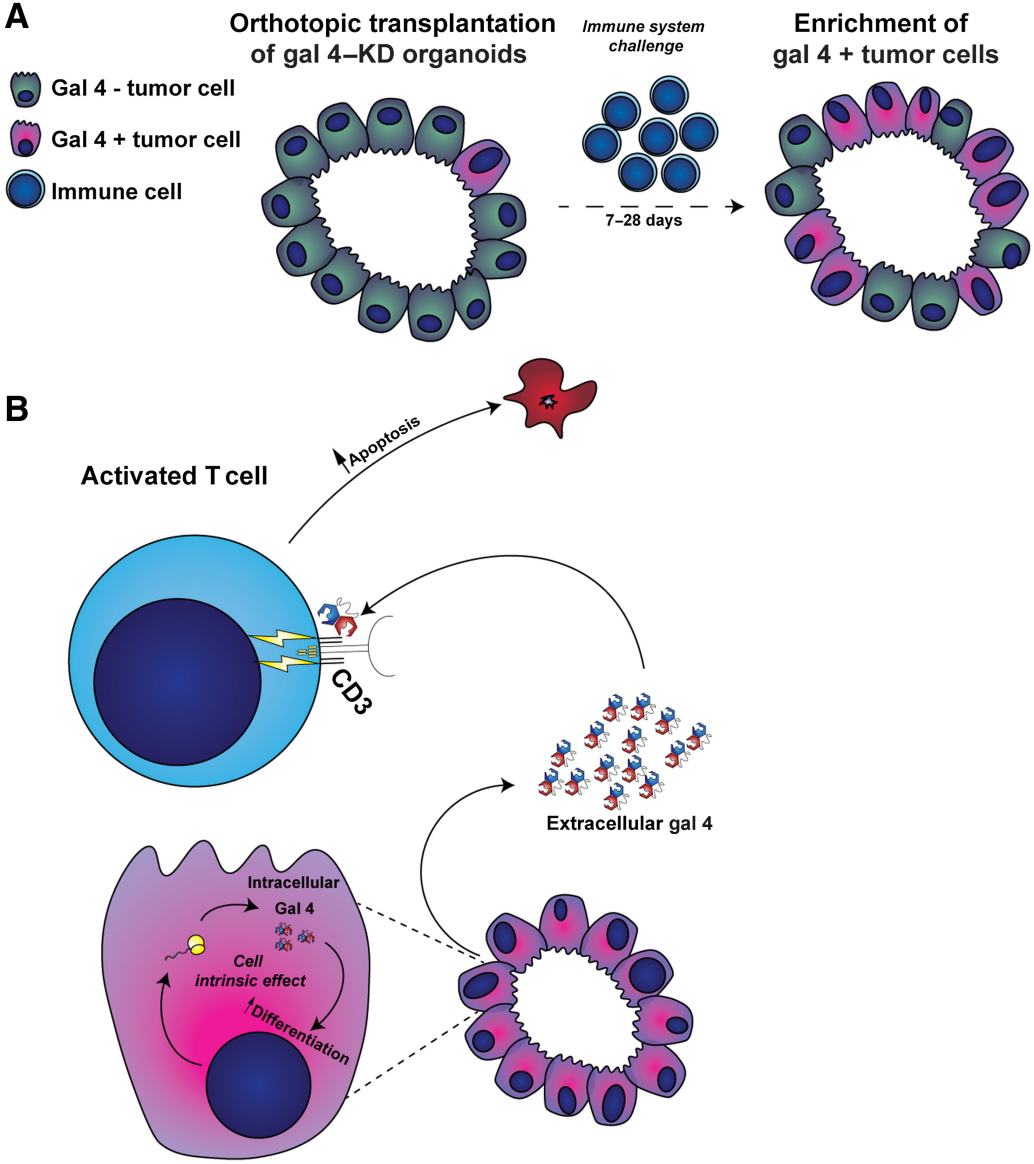
Proposed model of action of galectin 4 in pancreatic cancer. **A,** Proposed model of gal 4 recurrence in short-term orthotopic transplants of gal 4–KD organoid lines. Orthotopically transplanted gal 4–expressing cells expand at a greater rate when challenged by immune cells compared with gal 4–negative cells. **B,** Proposed model of the cell-intrinsic and extracellular effect of gal 4 on PDAC tumor cells and tumor-infiltrating T cells. Cancer cells produce gal 4, which has a cell-intrinsic effect, increasing differentiation of cancer cells. Gal 4 is secreted to the stroma, targets CD3 on the surface of T cells, and induces apoptosis in activated T cells, thereby suppressing antitumor immunity.

Our results also demonstrate that a complete KO of gal 4 in cancer cells enhances T-cell accumulation in tumors but does not affect killing of tumor cells, apparently due to effector T-cell exhaustion. The observed lack of effect could be due to the aggressive proliferation of the orthotopic transplantation model system. A complete KO of gal 4 also poorly recapitulates expression levels in human PDAC tumors. For this reason, we generated knockdown tumor organoid lines with reduced, but not fully eliminated, gal 4 expression. Orthotopic transplantation of gal 4–KD cell lines into immune competent C57/BL6J mice and immunodeficient RAG1^−/−^ mice demonstrated that the adaptive immune system was critical for increased survival. This is consistent with high levels of extracellular gal 4 in control tumors causing apoptosis of activated T cells, a phenomenon previously described in intestinal inflammation (15). The recurrence of gal 4 expression observed in survival experiments and short-term transplantation indicated an immune-mediated selection and expansion of the few cells expressing gal 4 at the onset of the experiment due to the survival advantage provided by gal 4 expression when challenged by adaptive immune cells (proposed mechanism illustrated in Fig. 7A). We did not, however, detect a difference in tumor weight in either 2D transplants or survival experiments with organoid cell lines. Differences in inflammation, as well as retention of liquid and tumor composition, could be factors affecting tumor weight. In organoid cell lines, we also detected gal 4 recurrence, increasing similarity between the tumors at endpoint.

Single-cell sequencing data from orthotopic transplants of scramble-control and gal 4–KD tumors showed differences in the proportion of immune cell populations with an increase in T cells and their activation, M1 macrophages polarization, and antigen-presenting cells, whereas the proportion of M2-immunosuppressive cells was reduced. A general increase in activated CTLs, M1 macrophages, and antigen-presenting immune cells was observed when gal 4 expression was reduced, which is known to be beneficial to patient survival (58–60).

The TCGA data set analysis showed significant associations between CD8 activity/cytolytic effect and gal 4 expression. Though, these types of data should be interpreted with caution because analysis of bulk sequencing data sets could be influenced by varying cellularity and tumor composition. However, from our single-cell data, we also detected increased activation in intratumoral T cells in gal 4–KD tumors, which supports the T-cell signature seen in the TCGA data and our other tumor models. The single-cell sequencing data also showed differences in CAF subtypes, indicating changes in the fibroblast compartment. This can be either through direct effects of gal 4 on fibroblasts, or indirectly through the effect of gal 4 on immune cells. We detected a higher proportion of myCAFS and significantly upregulated gene expression of myCAF markers in gal 4–KD tumors compared with scramble-control tumors. In contrast, we found fewer iCAFs in gal 4–KD conditions and significantly downregulated iCAF markers. A trend toward less apCAFs in gal 4–KD tumors was also detected, and it has been suggested that apCAFs have immunosuppressive features (61).

The *ex vivo* data from our coculture systems demonstrate that secretion of gal 4 by tumor cells can induce apoptosis in T cells. This is consistent with findings in inflammatory bowel disease by Paclik and colleagues (15), wherein they also show that gal 4 can bind to CD3 and induce calpain-dependent apoptosis in human T cells. Some glycosylated amino acid residues in CD3δ were highly conserved between species. Because gal 4 is a lectin and binds glycosylated residues (16, 17), we considered these sites to be likely binding sites for gal 4 on CD3. We demonstrated binding, including cross-species binding, between CD3δ/ε and gal 4, which was reduced after N-linked glycosidase treatment. To verify that this effect also translated to a biological system, we tested competitive inhibition with CD3 chains in an *ex vivo* model. The *ex vivo* model showed that blocking gal 4 binding to CD3, using soluble CD3 chains, removed the difference in apoptosis observed between gal 4–producing and –KO cell lines, indicating that the effect we observed is mediated through interaction with CD3. There is now a great body of evidence (62) that suggests that treatment with CD3-signal modulating antibodies can induce apoptosis (63) or anergy (64) in T cells. We hypothesize that gal 4 binding modulates CD3–TCR signaling, similar to these CD3 antibodies (63). The use of anti-CD3 does not affect FOXP3^+^ Tregs (65), which is in line with our data from orthotopic transplantations of R26 control and gal 4–KO lines. We saw an increased abundance of CD4^+^ and CD8^+^ cells, but no difference in FOXP3^+^ cells. Furthermore, anti-CD3 antibody therapy is used to induce tolerance in T cells (62), which supports the idea of gal 4 promoting tolerance to food antigens in the GI tract. This potential involvement of gal 4 in tolerance is also supported by the exclusive expression pattern of gal 4 throughout the GI tract that is observed under normal physiological conditions.

In this study, we identify gal 4 as novel immunomodulatory factor in PDAC. In contrast with other potential immunomodulatory factors in PDAC like CXCL12 (66) or Galectin 1 (67), which have been shown to be produced by stromal cells, gal 4 is produced by tumor cells and secreted to the ECM. The mechanism of secretion is unknown but has been linked to src kinase–mediated phosphorylation of gal 4 (68). Taken together with previous reports (19, 20, 69), our current findings demonstrate that gal 4 has a cancer cell–intrinsic role in cell differentiation and an extracellular role in T-cell exclusion (ref. 15; summarized in Fig. 7B). Our data further indicate that extracellular gal 4 is a potential target for immunomodulatory therapy, and inhibition of extracellular gal 4 could result in accumulation of activated T cells in the tumor. This would, in turn, sensitize PDAC, and potentially other cancers of the GI tract, to immune cell–mediated killing.

## Supplementary Material

Supplementary file 1 Metadata and single cell annotation for all 7677 cells in single cell dataset

Supplementary file 2 Top 30 markers of clusters in Immune subset of single cell data

Supplementary file 3 Top 30 markers of clusters in CAF subset of single cell data

Supplementary file 4 Lists of genes used to calculate signature scores for cytolytic activity, T cell activation and M1 vs M2 macrophage polarization in single cell data analysis

Supplementary file 5 Raw read count, normalized expression values (TPM) and sample metadata for bulk RNA sequencing data

Supplementary Figures 1-5 Supplementary Figures 1-5

Supplementary Figure Legends Legends for sup figures 1-5

Supplementary Tables Supplementary Tables
