# Supplementary figures and images for: Extracellular Galectin 4 Drives Immune Evasion and Promotes T-cell Apoptosis in Pancreatic Cancer
Source: Cancer Immunol Res. 2022 Dec 20;11(1):72–92. doi: 10.1158/2326-6066.CIR-21-1088 (PMC9808371; doi:10.1158/2326-6066.CIR-21-1088)

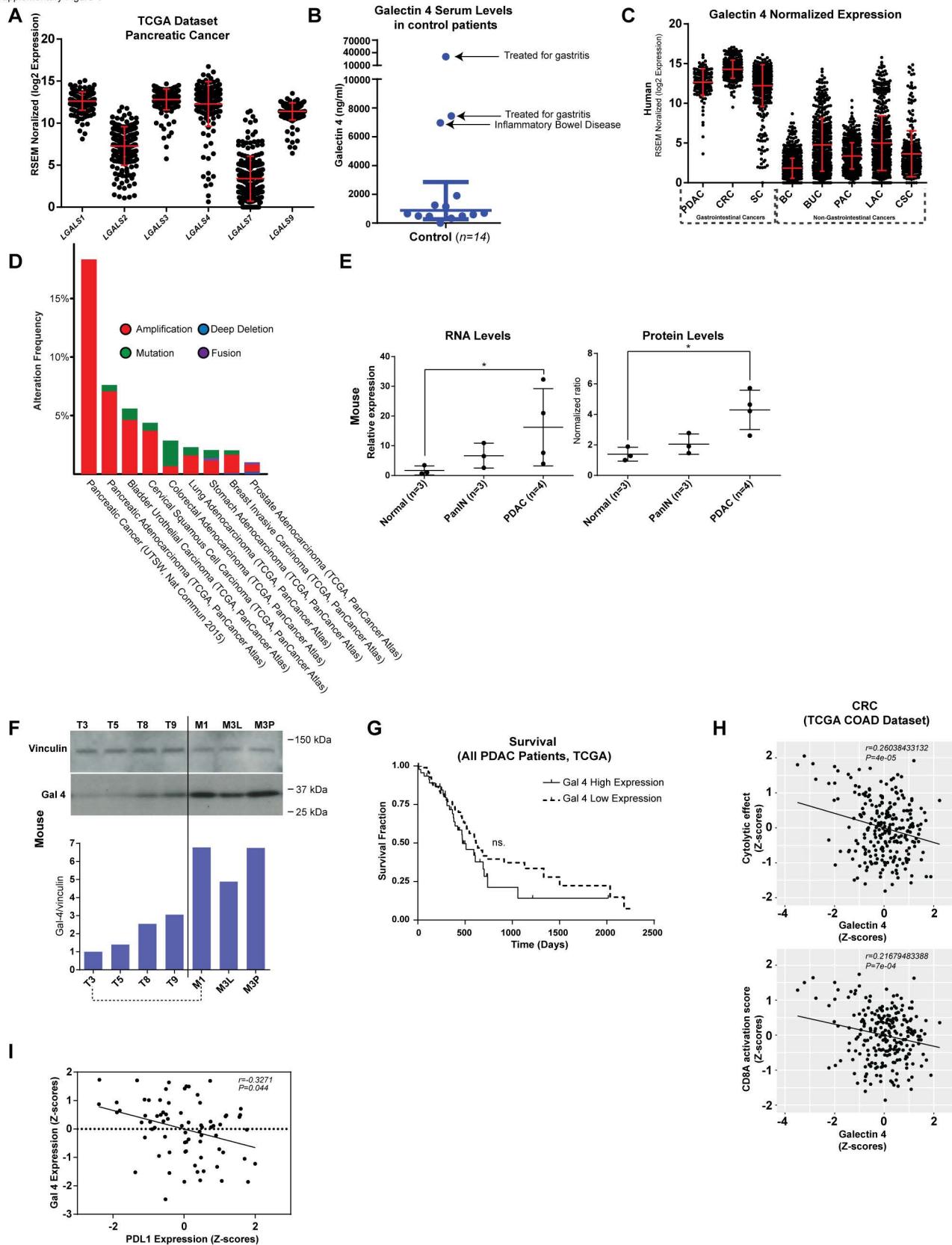

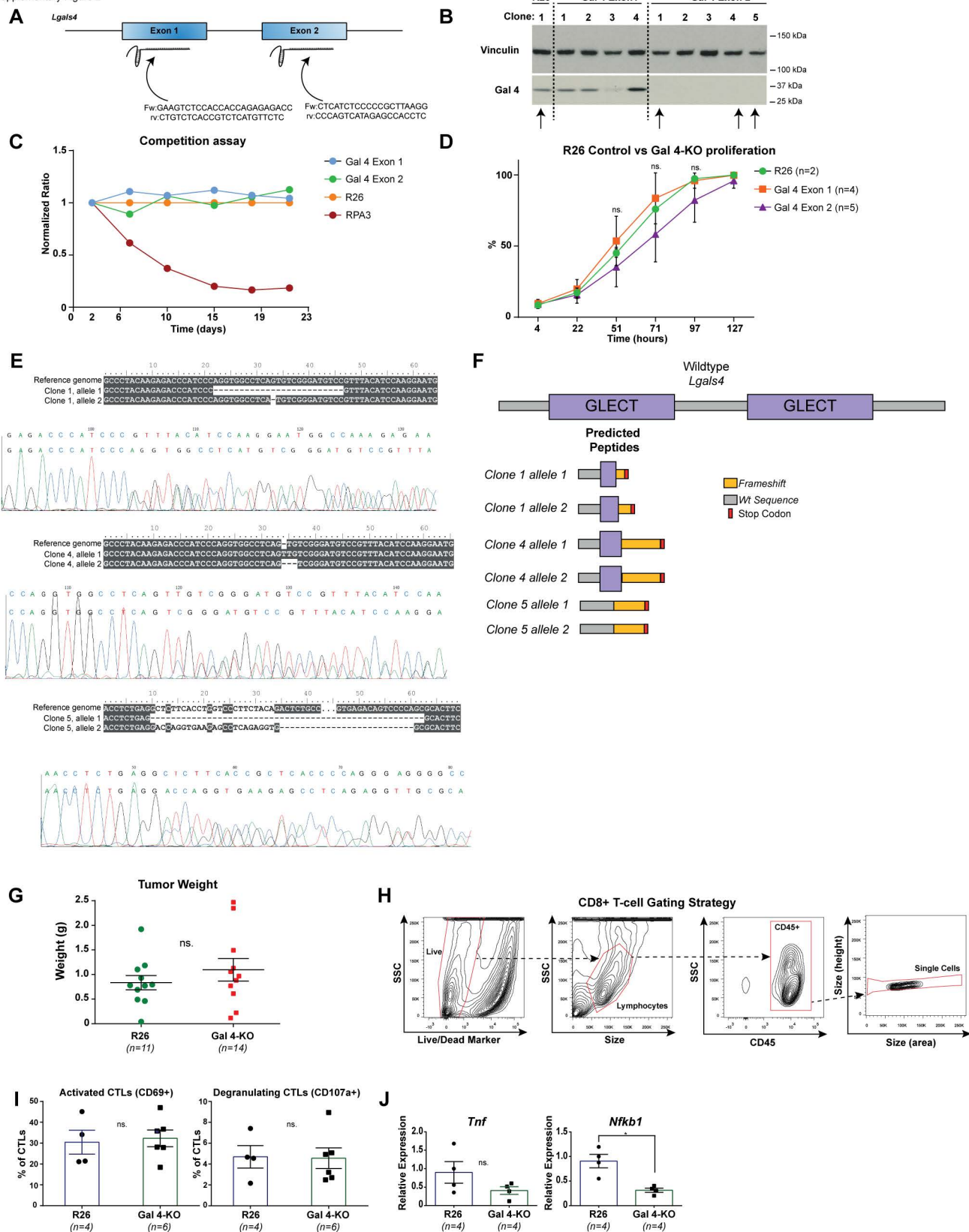

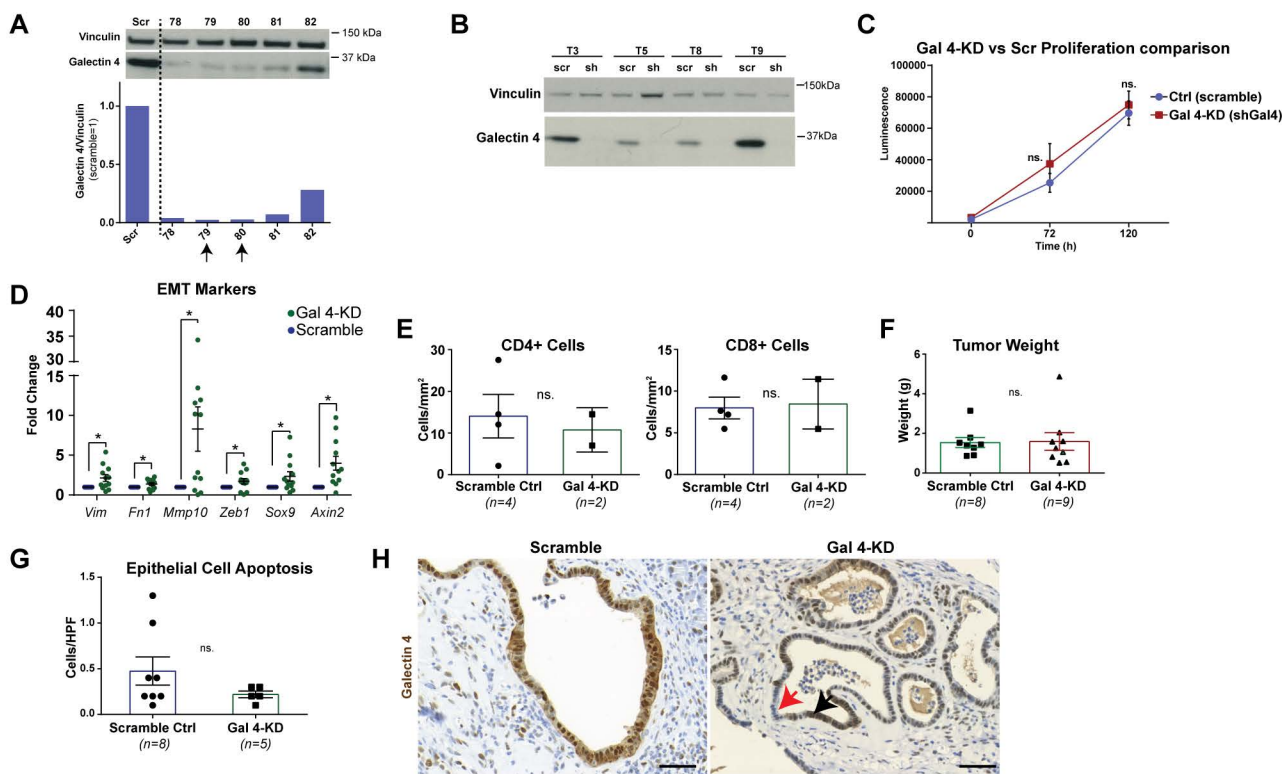

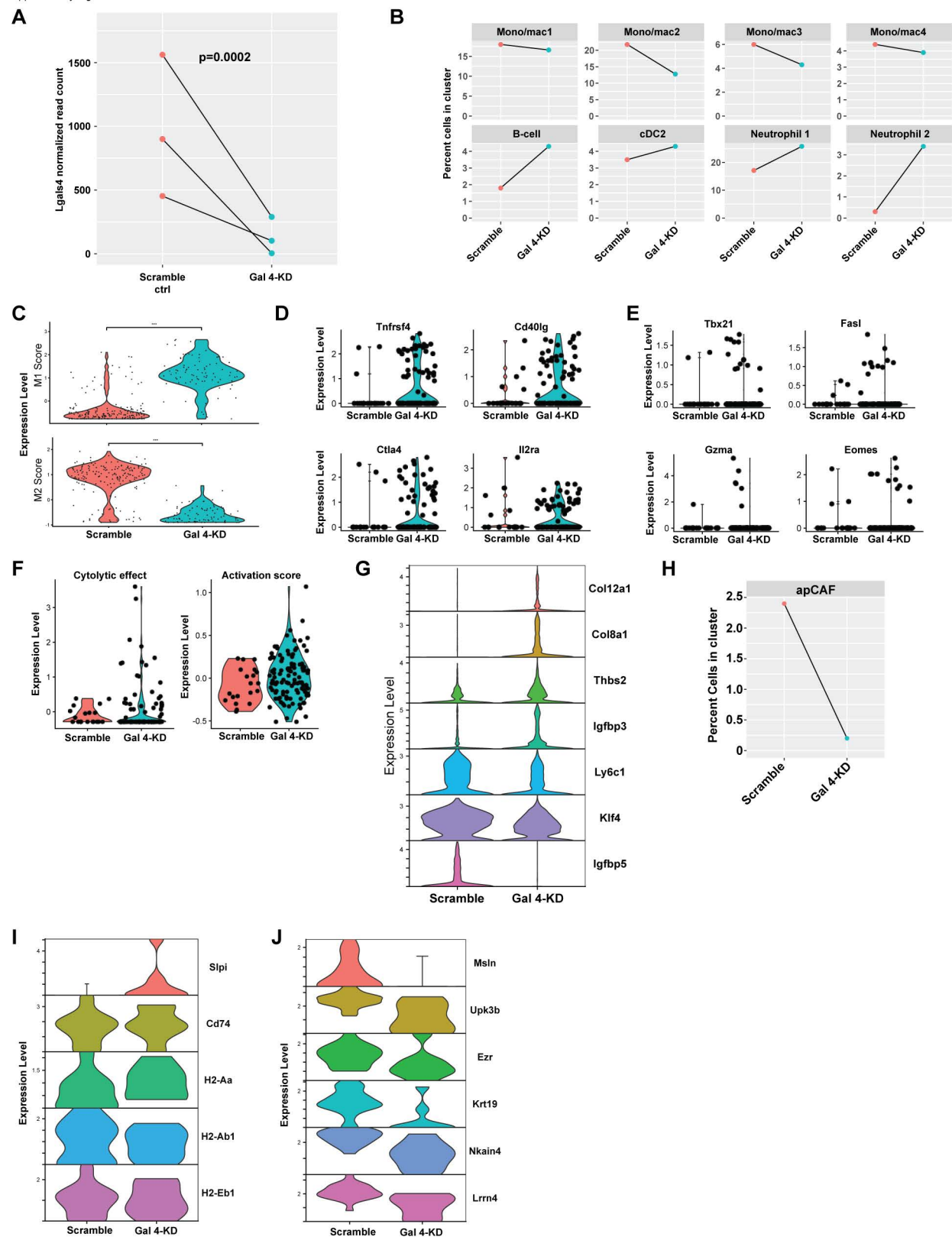

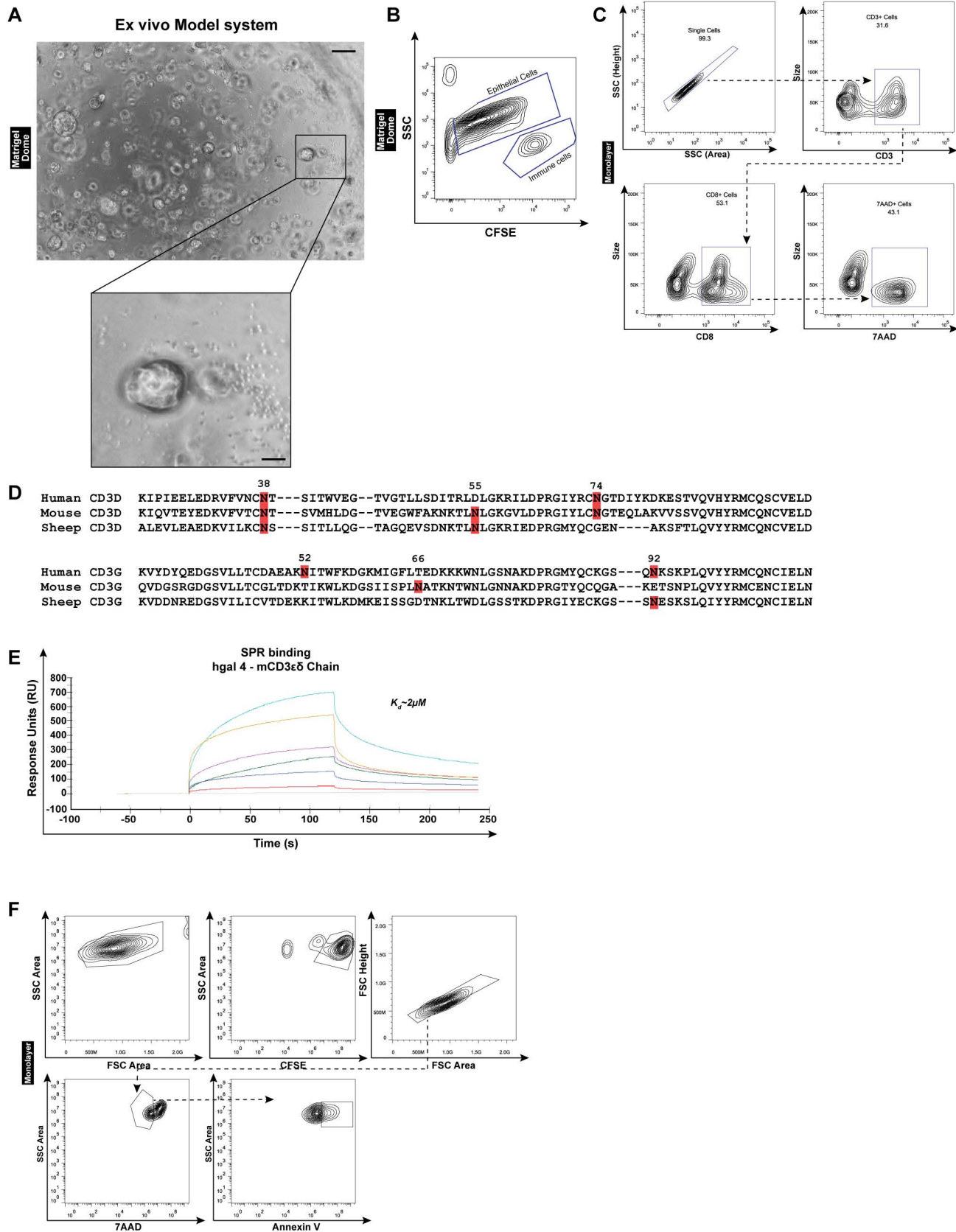

Supplement: Supplementary Figures 1-5 [file cir-21-1088_supplementary_figures_1-5_suppsf1-sf5.pdf]
