# Supplementary material for: Extracellular Galectin 4 Drives Immune Evasion and Promotes T-cell Apoptosis in Pancreatic Cancer
Source: Cancer Immunol Res. 2022 Dec 20;11(1):72–92. doi: 10.1158/2326-6066.CIR-21-1088 (PMC9808371; doi:10.1158/2326-6066.CIR-21-1088)
Supplement: Supplementary Figure Legends — Legends for sup figures 1-5 [file cir-21-1088_supplementary_figure_legends_suppsm6.pdf]

Supplementary Figure 1. Characterization of galectin 4 expression, genetic alterations, and co-regulation in organoids and published datasets

A RSEM normalized log2 RNA expression from TCGA dataset of human PDAC tumors (*n=183*) for the six galectin proteins detected in the mass spectrometry analysis on purified ECM [20]. *LGALS* are the corresponding gene names for the galectin proteins. Based on data generated by the TCGA Research Network: https://www.cancer.gov/tcga.

B Quantitative analysis (ELISA) of serum levels of gal 4 protein in controls. Arrows highlight controls excluded from further analysis because of inflammatory conditions in the GI tract. Error bars show mean with SEM.

C RSEM normalized log2 expression of gal 4 in TCGA datasets of pancreatic adenocarcinoma (PDAC, *n=151*), colon adenocarcinoma (CRC, *n=329*), stomach adenocarcinoma (SC, *n=450*), breast invasive carcinoma (BC, *n=1218*), bladder urothelial carcinoma (BUC, n=426), prostate adenocarcinoma (PAC, n=550), lung adenocarcinoma (LAC, n=576), and cervical squamous cell carcinoma (CSC, n=308).

D Gene alteration frequency data generated using cBioportal (*cbioportal.org*) comparing percentage levels of amplification, deep deletions, mutations, and fusions in datasets from different cancer types.

E RNA and protein expression levels of gal 4 in normal (N), PanIN (P) and tumor (T) murine organoid cell lines. Re-analysis of publicly available data from Boj *et al* [28]. RNA and protein levels normalized to one of the normal cell lines.

F Western blot and quantification(semiquantitative) of gal 4 protein levels in murine tumor (T) and metastatic (M) organoid lines. T3 and M1 are derived from the primary tumor and liver metastasis from the same KPC mouse (connected with dotted bracket). M3L and M3P are derived from a liver (L) metastasis and a peritoneal (P) metastasis, respectively, from the same KPC mouse. Vinculin is used as loading control.

G Kaplan-Meier survival curve calculated from the PDAC TCGA dataset with patients grouped into high *(n=48)* and low *(n=100)* gal 4 RNA expression (groups defined in materials and methods). ns = no statistically significant difference between the groups, calculated using Mantel-Cox test.

H Correlation dot plot showing gal 4 expression Z-scores versus cytolytic effect Z-scores (*top*) or CD8 activation scores (*bottom*) in the TCGA-COAD dataset of colorectal cancer *n=329*. Significance calculated using Pearson correlation.

I Correlation dot plot showing gal 4 expression Z-scores and PDL1 expression Z-scores in TCGA dataset of moderately differentiated PDAC tumors *(n=74)*. Significance calculated using Pearson correlation.

Supplementary Figure 2. Construction, validation, and functional assessment of galectin 4 deficient cell lines using CRISPR/CAS9

A Graphical representation of tested CRISPR sgRNA directed against exon 1 or exon 2 of the gal 4 gene.

B Western blot analysis of gal 4 and vinculin (loading control) on GFP sorted and expanded cell clones after transfection with guide RNA directed against the R26 locus (R26), exon 1 or exon 2 of the gal 4 gene. Arrows denote clones used in the following experiments described in figure 3.

C Drop out assay showing the fraction of infected cells (GFP+ cells) at different time points after infection with guide RNA directed against the R26 locus (R26), exon 1 of the gal 4 gene (Gal 4 Exon 1) or exon 2 of the gal 4 gene (Gal 4 Exon 2). Data normalized to the first time point and the R26 control. Guide RNA directed against Replication Protein A3 (RPA3), a gene necessary for replication, was used as positive control for the drop out assay.

D Proliferation assay measuring percentage covered area of cells at different time points for cell clones infected with guide RNA directed against the R26 locus, exon 1 of the gal 4 gene or exon 2 of the gal 4 gene (ns = no significant difference). Significance calculated using Mann-Whitney test.

E Sanger sequencing electropherogram of CRISPR/CAS9 modified Lgals4 in KPC 2D derived Gal 4-KO cell lines (Clone 1, 4, and 5) and alignments to the reference genome sequence of Lgals4.

F Predicted peptides of modified CRISPR/CAS9 sequences of Lgals4. GLECT indicates the functional galectin 4 carbohydrate recognition domains of the gal 4 protein.

G Dot plot showing tumor weight of R26 control and Gal 4-KO tumors (at the 28-day endpoint) orthotopically transplanted into immunocompetent *C57BL6/J* mice (ns = no significant difference).

H Gating strategy for determining CD8+ cell polarization in immune cells isolated from R26 control and Gal 4-KO tumors.

I Scatter plot with bar graph showing proportion of activated CD8+ T cells (*left*) and degranulating CD8+ T cells (*right*) isolated from R26 control and Gal 4-KO tumors.

J Scatter plot with bar graph showing relative expression of the inflammatory cytokine TNFα (*Tnf*), and inflammation related transcription factor *Nfkb1,* in R26 control (n=4) and Gal 4-KO (n=4) tumors. Normalized to a R26 control tumor. (ns. = no significant difference, * p=0.029). Significance calculated using Mann-Whitney test.

Supplementary Figure 3. Construction, validation, and functional assessment of organoid cell lines with reduced galectin 4 expression using RNA interference

A Western blot assessing knock-down efficacy of 5 different short hairpins directed against gal 4 (annotated as 78, 79, 80, 81, 82, catalog no. can be found in the material and method section) and to Scramble-hairpin controls (*upper panel*) with quantification (*lower panel*). Arrows denote the hairpins with the highest knockdown efficiency used for establishing organoids. Vinculin is used as loading control.

B Western blot depicting gal 4 expression in Gal 4-KD or Scramble-hairpin control tumor organoid lines (annotated as T3, T5, T8 and T9, T=tumor). Vinculin is used as loading control.

C CellTiter-Glo assay depicting the difference of proliferation of Gal 4-KD and scramble control organoid lines by luminescence measurements at 0h, 72 h and 120h. Gal 4-KD (n=4), scramble control (n=4), ns = no significant difference. Significance calculated using Mann-Whitney test.

D Scatter plot with bar graph depicting RT-qPCR quantification of genes linked to EMT in scramble controls (4 biological replicates, measured 3 times, n=12; normalized to 1) and Gal 4-KD cell lines (4 biological replicates, measured 3 times, n=12). * indicates significance, p-values: Vim = 0.015, Fn1 = 0.043, Mmp10 = 0.016, Zeb1 = 0.044, Sox9 = 0.033, and Axin2 = 0.002. Significance calculated using unpaired T-test.

E Scatter plot with bar graph showing quantification of CD4+ and CD8+ cell density in Gal 4-KD and scramble control tumors in IHC stained tissue from *C57BL6/J* mice of the survival cohort (ns = no significant difference).

F Dot plots showing tumor weight of scramble control and Gal 4-KD tumors (at the endpoint of survival experiment) orthotopically transplantated into immunocompetent *C57BL6/J* mice (ns = no significant difference).

G Scatter plot with bar graph showing quantification of apoptotic epithelial cells (CC3+) per High power field in Gal 4-KD and scramble control tumors in IHC stained tissue from *C57BL6/J* mice of the short-term cohort (ns = no significant difference).

H Representative IHC images of gal 4 staining of scramble control (left) and Gal 4-KD (right) tumors at 7-days (scramble controls) and 14-days (Gal 4-KD) post-transplantation. Arrows highlight cobblestone-like areas of gal 4 negative (red arrow) and positive cells (black arrow). Scale bar=50 µm.

Supplementary Figure 4. Single cell RNA-sequencing of dissociated orthotopic transplants of galectin 4 reduced organoid cell lines

A Line graph showing Lgals4 normalized read counts in bulk sequencing of Epcam+ cells of scramble control and Gal 4-KD cell lines using smartseq2. p = 0.0002 determined by a Wald test from Dseq2 package.

B Line graph showing changes of percent in cluster of immune cell subpopulations identified in single cell RNA sequencing. (Mono - monocytes, Mac – Macrophages, DC – dendritic cells)

C Violin plot showing differences in expression levels (log-normalized read count) between intratumoral M1 and M2 macrophages. p-values < 2*10^-16 calculated using Wilcoxon signed-rank test.

D Violin plot showing expression levels of T-cell activation markers between intra tumoral T cells from scramble control and Gal 4-KD tumors.

E Violin plot showing expression levels of T cell cytotoxic markers between intra tumoral T cells from scramble control and Gal 4-KD tumors.

F Violin plots showing differences in cytolytic and activation score, with genes from table S2, in intra-tumoral T cells.

G Violin plot showing significant differences in expression of myCAF and iCAF marker genes in scramble control and Gal 4-KD tumors. Adjusted p-values < 4*10^-8.

H Line graph showing percent in cluster differences of mesothelial derived apCAFs between scramble control and Gal 4-KD orthotopic transplants.

I Violin plots showing differences of MHC II pathway gene expression in apCAFs in scramble control and Gal 4-KD tumors.

J Violin plots of Mesothelial gene expression in apCAFs in scramble control and Gal 4-KD tumors.

Supplementary Figure 5. *Ex vivo* models of PDAC support gal 4 mediated T-cell apoptosis through CD3 delta

A Brightfield image of organoid *ex vivo* model showing organoids surrounded by small lymphocyte-like cells (scale bar 200 µm *top*, 50 µm *bottom*).

B Representative contour plot showing the gating strategy of CFSE labeled immune cells and epithelial cells used for Matrigel dome epithelial cell apoptosis analysis by 7AAD staining.

C Representative contour plot showing the gating strategy of T cells and CD8+ T cells added to R26 control or Gal 4-KO cell lines.

D Amino acid alignment of human, mouse and sheep CD3 delta chain. Conserved N (asparagine) residues, with N-linked glycosylations highlighted in *red*.

E SPR bindning graph showing binding between recombinant human gal 4 (hgal 4) and recombinant mouse CD3 epsilon/delta (mCD3εδ) chain.

F Contour plot showing gating strategy for AnnV+ CD8+ T cells cells from *ex vivo* monolayer cell experiments using R26 control or Gal 4-KO cell lines with BSA and CD3 chains in 2D *ex vivo* model.
