# Supplementary material for: Extracellular Galectin 4 Drives Immune Evasion and Promotes T-cell Apoptosis in Pancreatic Cancer
Source: Cancer Immunol Res. 2022 Dec 20;11(1):72–92. doi: 10.1158/2326-6066.CIR-21-1088 (PMC9808371; doi:10.1158/2326-6066.CIR-21-1088)
Supplement: Supplementary Tables [file cir-21-1088_supplementary_tables_suppst1.pdf]

Supplementary table 1 (Table S1)

**Antibody (target)**	**Manufacturer**	**Dilution**	**Assay**
CD45	Thermo 69-0451-82	1:100	FACS
CD45	Thermo 48-0451-82	1:100	FACS
CD3	Biolegend 100204	1:100	FACS
CD4	Biolegend 100516	1:100	FACS
CD8a	BD 563068	1:100	FACS
PD-1	Biolegend 109103	1:50	FACS
TIM-3	Biolegend 134009	1:50	FACS
CD326	Thermo 12-5791-82	1:100	FACS
CD69	Thermo 47-0691-82	1:50	FACS
CD107a	Thermo MA5-28671	1:50	FACS
CD4	Abcam ab183685	1:1000	IHC
CD8a	Abcam EPR20305	1:2000	IHC
FOXP3	CST 12653S	1:500	IHC
Cleaved Caspase 3	CST 9579S	1:400	IHC
Gal4 (E-2)	Santa cruz sc-271209	1:200	IHC
Gal4	NBP2-48605	1:100	IHC/WB
Gal4	Santa cruz sc-19286	1:200	WB
Vinculin	Merck, V4139	1:1000	WB

Supplementary table 2 (Table S2)

**Cytolytic Effect**	**CD8 Activation Score**
EOMES	*CD69*
TBX21	*CCR7*
GZMB	*CD27*
PRF1	*BTLA*
FASLG	*CD40LG*
GZMH	*IL2RA*
GZMA	*CD3E*
	CD47
	EOMES
	GNLY
	GZMA
	GZMB
	PRF1
	IFNG
	CD8A
	CD8B
	FASLG
	LAMP1
	LAG3
	CTLA4
	HLA-DRA
	TNFRSF4
	ICOS
	TNFRSF9
	TNFRSF18

Supplementary table 3 (Table S3)

**Primers**	**forward Sequence**	**Reverse Sequence**
Nfkb1	TCTGGCACAGAAGTTGGGTC	GGTACCCCCAGAGACCTCAT
Tnf	CCACCACGCTCTTCTGTCTAC	AGGGTCTGGGCCATAGAACT
FN1	GGTCTACGGCAGTTGTCACA	GCAACGTGCTATGACGATGG
VIM	GAAATTGCAGGAGGAGATGC	TCCACTTTCCGTTCAAGGTC
Zeb1	TGAGCACACAGGTAAGAGGCC	GGCTTTTCCCCAGAGTGCA
Sox9	CATTCCTCCTCCGGCATGAG	TCAACTTTGCCAGCTTGCAC
Mmp10	GGCCCACTCTTCCTTCAGAC	TTCATTTCCTCGGACTGCCC
Axin2	TCAAGGAGCAGCTCAGCAAA	CCAGGATCCTGCCTTCGTAC
